# Alternate splicing of transcripts shape macrophage response to *Mycobacterium tuberculosis* infection

**DOI:** 10.1371/journal.ppat.1006236

**Published:** 2017-03-03

**Authors:** Haroon Kalam, Mary F. Fontana, Dhiraj Kumar

**Affiliations:** Cellular Immunology Group, International Centre for Genetic Engineering and Biotechnology, Aruna Asaf Ali Marg, New Delhi, India; New Jersey Medical School, UNITED STATES

## Abstract

Transcriptional reprogramming of macrophages upon *Mycobacterium tuberculosis* (*Mtb*) infection is widely studied; however, the significance of alternate splicing (AS) in shaping cellular responses to mycobacterial infections is not yet appreciated. Alternate splicing can influence transcript stability or structure, function and localization of corresponding proteins thereby altering protein stoichiometry and physiological consequences. Using comprehensive analysis of a time-series RNA-seq data obtained from human macrophages infected with virulent or avirulent strains of *Mtb*, we show extensive remodeling of alternate splicing in macrophage transcriptome. The global nature of this regulation was evident since genes belonging to functional classes like trafficking, immune response, autophagy, redox and metabolism showed marked departure in the pattern of splicing in the infected macrophages. The systemic perturbation of splicing machinery in the infected macrophages was apparent as genes involved at different stages of spliceosome assembly were also regulated at the splicing level. Curiously there was a considerable increase in the expression of truncated/non-translatable variants of several genes, specifically upon virulent infections. Increased expression of truncated transcripts correlated with a decline in the corresponding protein levels. We verified the physiological relevance for one such candidate gene RAB8B; whose truncated variant gets enriched in H37Rv infected cells. Upon tweaking relative abundance of longer or shorter variants of RAB8B transcripts by specialized transduction, mycobacterial targeting to lysosomes could be promoted or blocked respectively, which also resulted in corresponding changes in the bacterial survival. Our results show RAB8B recruitment to the mycobacterial phagosomes is required for phagosome maturation. Thus the abundance of truncated RAB8B variant helps virulent *Mtb* survival by limiting the RAB8B levels in the cells, a mechanism which we subsequently verified in human primary macrophages. Taken together we demonstrate alternate splicing as a new locus of intervention by *Mtb* and provide attractive alternative to exploit for novel drug targets against *Mtb*.

## Author summary

Eukaryotic gene expression is a complex process where several intermediary processing steps of transcripts are required before translation can take place. Change in gene expression is a fundamental means through which cells adapt to environmental cues or stimuli like infections. The professional phagocytes macrophages are known to undergo drastic alteration in gene expression upon inflammatory stimuli or infection with pathogens like *Mycobacterium tuberculosis*. However, whether the alterations in gene expression are also influenced by possible perturbations of one or more of the intermediate steps in RNA processing is not known. Here taking advantage of next-generation RNA sequencing approach, followed by robust computational analysis, we show infection of macrophages by *Mycobacterium tuberculosis* results in massive alterations in the splicing pattern in the host. Since alternate splicing can influence transcript stability, stability of the translated products, loss/gain of function, interacting partners and sub-cellular localization, its implications on host response to infection could be overwhelming. We subsequently performed targeted experiments to confirm that alternate spliced variants of host genes indeed helped infecting *Mtb* strains to survive better within the macrophages. Regulators of splicing, either from host and/or pathogen, therefore, constitute an attractive set of targets to develop novel therapeutic strategies to control tuberculosis.

## Introduction

Infection of host macrophages with *Mycobacterium tuberculosis* results in significant alterations in the macrophage physiology. This includes altered regulation of common microbicidal processes like phagosome maturation, autophagy, apoptosis and other innate immune functions [1, 2]. While the early responses like phagosome maturation are regulated through the perturbation of immediate signaling events like activation of signaling through PI3kinases and Ca^2+^ dependent kinases (PKCs, CAMKII) or pro-survival pathways [3, 4], long term shaping of the macrophage response largely depends on the modulation of transcriptional machinery resulting in a modified protein complement and physiology of the cells [5].

In order to track the changes in gene expression in the macrophages upon *Mtb* infections, several studies in the past reported microarray analysis of total RNA from infected macrophages, providing a great deal of information on the shaping of cellular responses upon infection [6–14]. Thus it was reported that genes involved in immune regulation, inflammation, metabolism and cell survival were differentially regulated upon *Mtb* infections [6, 11, 12]. In one such study, we previously showed distinct expression and activity of the tyrosine kinase Src in the survival of virulent *Mtb* strain H37Rv [5]. While the global expression analysis using microarray does provide details of cellular responses, an apparent non-correlation between gene expression and protein translation in the eukaryotes has always confounded the inferences drawn from these analyses [15–17]. The potential reasons for this discrepancy include a longer time lag in the eukaryotes between transcription and translation as well as the involvement of complex post-transcriptional mechanisms like splicing, poly-adenylation, RNA-editing, etc. [17].

Splicing incorporates lots of heterogeneity in the transcripts from a single gene by selectively retaining or excluding specific exons in the final processed mRNA. This leads to the generation of several alternately spliced variants of a single gene [18, 19]. The alternate spliced variants of a transcript may have different structures, different functions, different sub-cellular localizations and stability owing to presence or absence of specific exons coding for specific protein modules [20]. Many of the alternate spliced variants do not get translated for reasons like presence of premature stop codons in each of the reading frames. The non-translatable transcripts may eventually get degraded through a process called non-sense mediated decay (NMD) [21]. Other truncated transcripts may get translated into a truncated protein with significant alterations in activity, binding, localization, etc. The role of alternate splicing in regulating the immune responses of cells is increasingly getting recognized. A series of studies show alternate splicing as a common mechanism that influences several molecules of the TLR signaling pathway and thereby regulates the inflammatory responses of the cell [22, 23]. Several viral pathogens are known to alter AS events in the cells to facilitate viral replication and control cell cycle [24, 25]. Splicing of eukaryotic transcripts occurs through a dynamic multi-step macro-molecular complex called spliceosome, considered as one of the most complex molecular machineries in the cell [26]. At different steps of spliceosome assembly and progression one or more of snRNPs (small nuclear ribonucleoproteins) like U1, U2, U4/U6 and U5 are involved each consisting of one or more snRNAs complexed with a vast number of accessory proteins. Their recruitment, catalysis and release are highly regulated to achieve high specificity of splicing as well as high flexibility to accommodate alternate splicing [27, 28]. Involvement of a large set of proteins and multi-step regulation renders spliceosome functions vulnerable to cues where a large number of genes get differentially regulated. Intriguingly, while the global changes in gene expression upon infection of macrophages with Mtb are extensively studied there is no clear description of whether spliceosome components too get differentially regulated in the infected macrophages.

The 3’UTR of the mammalian transcripts are yet another regulatory feature, mostly controlling the stability of the transcript [29, 30]. The 3’UTR contains several potential miRNA binding targets and therefore based on the utilization of a proximal or distal poly-adenylation site a transcript may specifically exclude or include a particular miRNA site respectively [30]. The significance of alternate poly-adenylation has been shown in the case of cancers where individuals showed large variations in the poly-A site utilization [30–32]. Recently an alternate 3’UTR was shown to serve as a scaffold to regulate membrane protein localization [33]. Regulation of miRNA upon *Mtb* infections is an intense area of investigation. Several studies in the past have shown specific miRNA expression upon *Mtb* infection [34–38]. It is therefore very likely that the cell could have evolved the APA strategy to counter the miRNA-mediated decay in the infected macrophages as well.

Here we followed the time-resolved transcriptome data of THP-1 macrophages that were infected with H37Rv (virulent) or H37Ra (non-virulent) strains of *Mycobacterium tuberculosis*. We report extensive alternate splicing and alternate poly-adenylation events at the global scale in infected macrophages. Importantly, the transcript variants formed due to these events significantly contributed in deciding the fate of cellular response to infections.

## Results

### Complete transcriptome of *Mtb-*infected THP-1 macrophages

**Fig 1:**
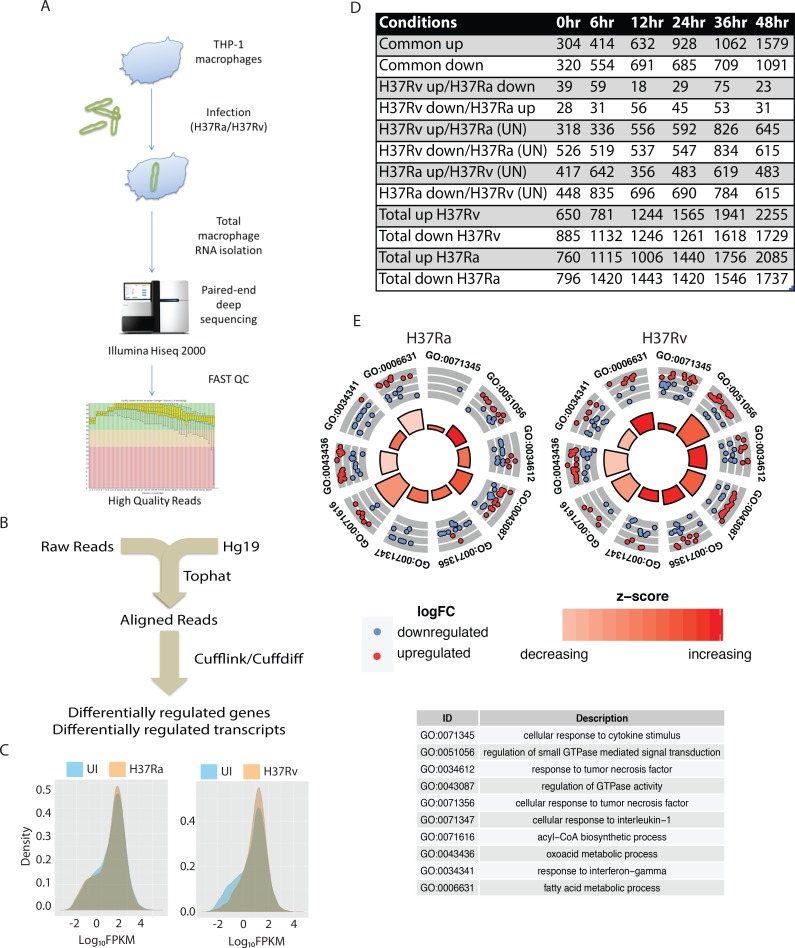
RNA-seq analysis of THP-1 macrophages infected with *Mycobacterium tuberculosis*. (A) Flow-chart of the RNA-seq experiments. PMA differentiated THP-1 macrophages were infected with H37Ra or H37Rv for different time points, total host RNA was isolated and sent for RNA-seq. (B) Strategy assembly for the analysis of raw reads obtained from RNA-seq experiment (C) Density plots overlaying distribution of gene level FPKM in UI, H37Ra or H37Rv infected THP-1 macrophages. The data shown is for 36 hours time points. (D) Differentially expressed genes between H37Ra and H37Rv infected macrophages obtained through gene level quantification of the data (up: two fold increase in expression; down: two fold decrease in expression; UN: no change in expression) (E) Gene ontology (GO) enrichment analysis of genes differentially expressed in H37Ra and H37Rv infected macrophages at 48 hours post-infection. Select GO classes are plotted here to show the effect on genes belonging to the two classes and the statistical significance (z-score). Every dot in the plot corresponds to one gene, while the color of dots show either up (red) or down (blue) regulation. Height and color of inner circle bars correspond to the p-value and significance.

Total RNA isolated from H37Ra and H37Rv infected THP-1 macrophages at 0, 6, 12, 24, 36 and 48 hours post-infection were used to make cDNA libraries followed by sequencing using Illumina Hiseq2000 platform (see methods for detail). The experimental set-up is schematically shown in Fig 1A. Quality control for the paired end raw sequence data set was performed using FASTQC kit. The reads obtained were of very high quality as more than 95% of reads across the conditions crossed the Phred score of Q20 while more than 88% of reads were above Q30 threshold (S1A Table). A score of Q20 corresponds to incorrect base call at 1in 100 while the score of Q30 means incorrect call at 1 in 1000. These values correspond to overall base call accuracy of 99% (Q20) and 99.9% (Q30) respectively. Reads with a score higher than Q30 were taken further for downstream analysis. The downstream analysis flowchart is shown in Fig 1B. For each sample, approximately 180 million paired-end reads of 101 bp was used for genome-guided alignment using Tuxedo pipeline. Alignment of raw reads on human genome build Hg19 was carried out using splicing aware Tophat aligner [39]. For each sample, more than 70% of reads aligned on Hg19 (S1B Table). Of the aligned reads, ~3% aligned to intronic regions, ~2% aligned to intergenic regions and the remaining ~94% aligned to exons. The absolute quantitation (fragments per kilobase per million reads or FPKM) of genes and transcripts and their differential regulation as compared to the uninfected control was obtained using Cufflink and Cuffdiff package [39, 40]. The analysis through Cufflink-Cuffdiff package also provides statistical measures for identifying every regulated gene. The entire list of regulated genes across the time points in both H37Ra and H37Rv infected cases is provided in S2 Table. We also characterized a few global properties of expression profiling before analyzing the genes that were differentially expressed. First, the dispersion analysis of reads aligning to the genome revealed an absence of any sample specific bias in alignment (S1A Fig). Further, we tested deviation of individual gene expression around the median of the particular sample. All the 12 samples showed a nearly identical median; however there were some infection and time point specific alterations in the deviation, like more down-regulated genes in H37Ra infected macrophages at 0 and 6 hours but more down-regulated genes in H37Rv infected macrophages at 12, 24 and 36 hours post-infection (S1B Fig).

The density distribution plot, shown here for 36-hour time point, shows relative differences in the gene level expression between H37Ra and H37Rv infected cells with respect to uninfected cells (Fig 1C). Similar plots for each of the time points are shown in supplemental S2 Fig. Between H37Ra and H37Rv infected macrophages we got several unique and overlapping sets of genes showing specific regulation across different time points. Fig 1D comprises a list of numbers of all such uniquely or commonly regulated genes across the points. The comparison groups included identifying genes that were commonly up or down regulated (rows 1 and 2 in Fig 1D), genes which show exactly contrasting pattern like up in H37Rv and down H37Ra or vice versa (rows 3 and 4 in Fig 1D), cases where genes are up or down regulated in H37Rv and un-regulated in H37Ra and vice-versa (rows 5, 6, 7 and 8 in Fig 1D) and total number of genes showing up or down regulated pattern in one case irrespective of the strain case (rows 9, 10, 11 and 12 in Fig 1D). This analysis was performed at each of the time points (Fig 1D). A few simple observations emerged through this analysis, e.g. there were more common up-regulated genes than down-regulated ones. Secondly, the number of genes showing exactly contrasting expression between H37Ra and H37Rv infected cells was very small (<100 throughout except for 36 hours time point, rows 3 and 4 combined in Fig 1D). The functional class analysis of these differentially regulated genes followed expected patterns, as shown for 48 hours time point in Fig 1E and remaining time points in S3 Table. Gene enrichment analysis revealed significant enrichment of genes belonging to metabolism, gene regulation, trafficking, immune, inflammation and chemokine/cytokine signaling suggesting, expectedly, massive perturbation of macrophage innate immune function. The functional classes overlapped with our previously published microarray experiments [5].

### Transcript level expression profile grossly differs from corresponding gene level profiles

**Fig 2:**
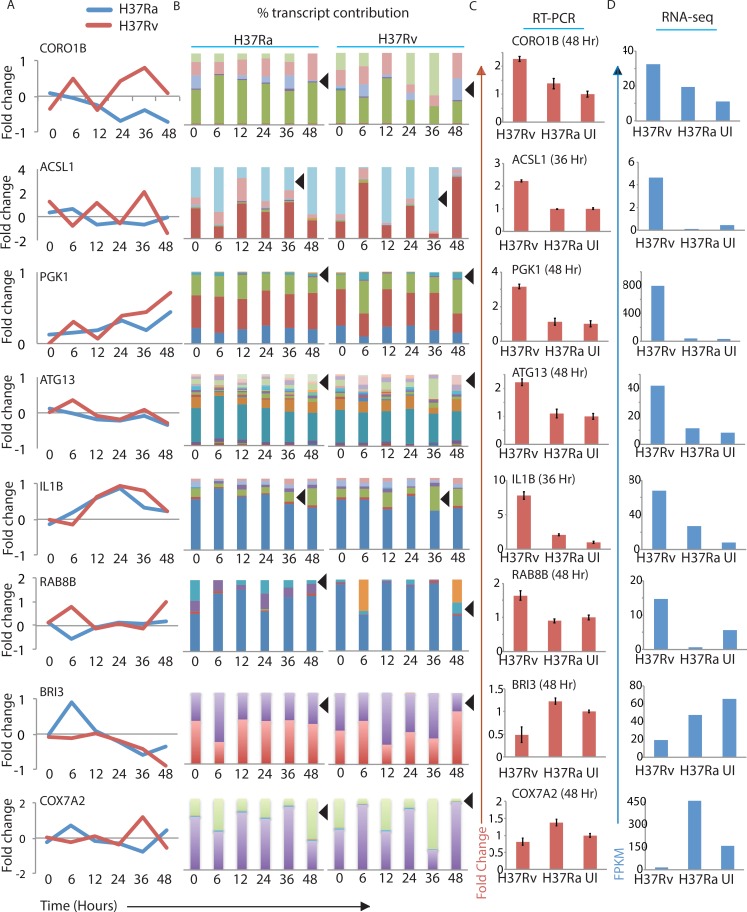
Gene level quantification differs considerably from isoform level quantification. (A) Fold change in the expression was calculated at gene level as well as isoform levels. Gene level changes in expression across the time points for eight genes (as mentioned) in H37Ra (blue) or H37Rv (red) infected macrophages are plotted as line graph. (B) Expression values at isoform levels for the respective genes under H37Ra (left) or H37Rv (right) infected conditions were normalized to 100% and contribution from different isoforms were plotted as different sub-bars. The black arrowheads indicate the transcript and the time point that was selected to validate the RNA-seq data using Q-PCR (shown at the right in Fig 2C). (C) cDNAs from uninfected (UI), H37Rv infected (H37Rv) and H37Ra infected (H37Ra) macrophages at the mentioned time points were analyzed by quantitative PCR using isoform specific primers (see methods). Plots represent fold-change with respect to uninfected control (Values ±S.D.). (D) Corresponding FPKM values for the transcripts at the time points validated in 2C were plotted separately here for the ease of comparison.

We were keen to understand transcript level expression pattern in the infected macrophages. Most of the genes in the human genome have known transcript variants and isoforms. We modified the transcript-specific GTF file (see methods) and followed the Cufflink-Cuffdiff package to obtain transcript-specific expression data. We first checked there was no sample specific bias in the dispersion of reads alignment at the transcript level. We also compared transcript level distribution of expression in each of the samples with respect to the uninfected control. All samples showed near normal distribution of transcript level expression (S3A Fig). The whole list of isoform-specific expression across the groups and time points is provided in supplemental S4 Table. At 36 hours, H37Rv infected cells transcript expression distinctly diverged from the uninfected cells, however, continued to show normal distribution suggesting significant regulations at this time point (S3A Fig). We were curious to see whether transcript level expression profile differed from the pattern observed at the gene level. We checked transcript expression for several genes like *CORO1B*, *ACSL1*, *PGK1*, *ATG13*, *IL1B*, *RAB8B*, *BRI3* and *COX7A2* (Fig 2A). The selection of genes was driven by empirical observation of differential transcript expression as well as due to their easy association with innate immune regulation and cellular responses to *Mtb* or other infections [41–44]. The most common observation was that different transcripts of a given gene showed wide variations in terms of expression and greatly differed from the patterns observed at the corresponding gene level (Fig 2B). For most of the cases, the expression of transcripts was more contrasting and prominent between H37Ra and H37Rv infected samples at later time points specifically at 24 and 36 hours post-infection, as discussed in the next section. In cDNAs prepared from independent experiments, we verified expression patterns observed at the transcript levels for select isoforms of each of the genes discussed in Fig 2A through Q-PCR analysis. The selection of transcript for validations was mainly driven by two factors: it should show large differences at FPKM level, which could allow them to be picked up by RTPCR analysis. Secondly, we focused more on shorter transcripts of each gene. The significance of shorter transcripts is discussed in more detail in subsequent results sections. Thus we picked ENST00000545736, ENST00000505492, ENST00000491291, ENST00000579280, ENST00000496280, ENST00000558990, ENST00000456357 and ENST00000472311 for *CORO1B*, *ACSL1*, *PGK1*, *ATG13*, *IL1B*, *RAB8B*, *BRI3* and *COX7A2* respectively (marked as arrowheads in Fig 2B). The expression pattern between uninfected, H37Ra infected and H37Rv infected largely matched between the independent Q-PCR experiments (Fig 2C) and RNA-seq data (Fig 2D). While the variation at the isoform level expression was an interesting observation, it also raised questions about the suitability of earlier approaches using microarray. Different isoforms of a gene vary in their functionality due to reasons like inclusion/exclusion of certain exons, stability of the transcript and differential targeting [20]. Variations in the isoform level expression arise due to differential alternate splicing (AS) events and therefore we decided to analyze the global pattern of AS upon infection.

### Differential transcript expression highlights massive alternate splicing in infected macrophages

**Fig 3:**
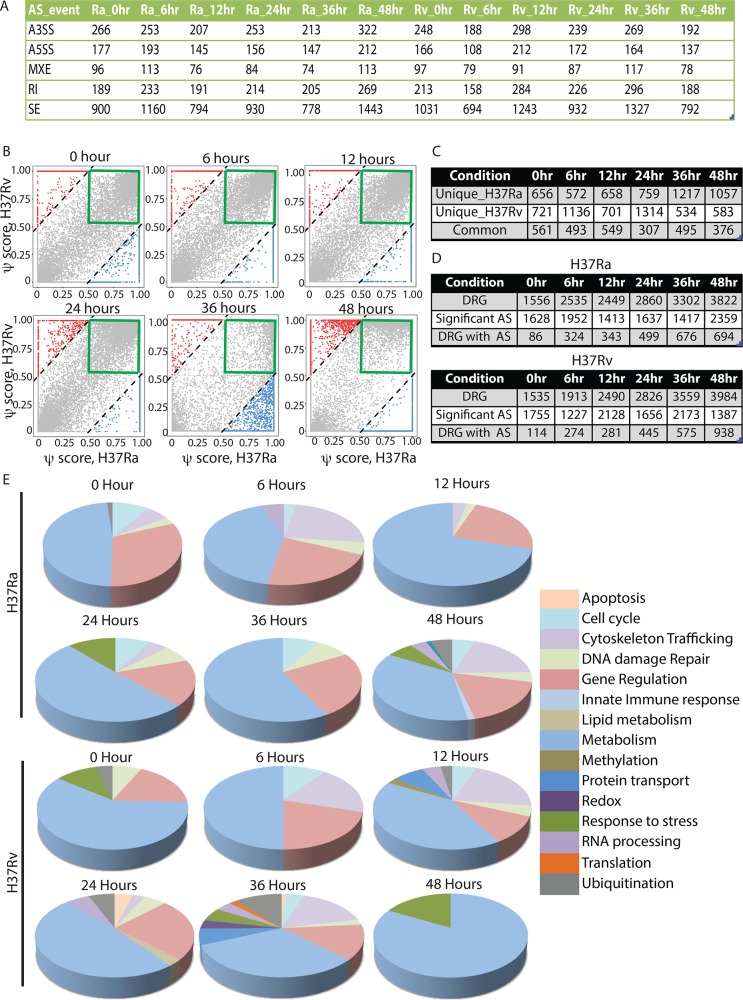
Estimation of alternate splicing events reveal infection and strain specific regulation. (A) Number of alternate splicing events where psi-score compared to the uninfected control was higher than 0.5 across each of the groups and time points is shown here. A3SS: alternate 3’ splice site, A5SS: alternate 5’ splice site, MXE: mutually exclusive exons, RI: retained introns, SE: skipped exons. (B) Comparative dot-plots for isoform specific psi-scores for H37Ra and H37Rv infected macrophages plotted for each of the six time points. Every dot in the plot represents one gene. The diagonal lines mark the regions beyond which transcripts had higher psi-score in H37Rv infected cells by 0.5 or more with respect to H37Ra infected cells (red) or in H37Ra infected cells by 0.5 or more with respect to H37Rv infected cells (blue). The green boxes highlight those cases where psi-score was higher than 0.5 in both H37Ra and H37Rv infected cells. (C) A comparative list of number of genes undergoing AS that were unique to H37Ra, H37Rv infection and those that were common to both the infection conditions at each of the time points. (D) Comparative list showing number of differentially regulated genes (DRGs), number of significant AS cases and number of DRGs showing AS across the time-points in H37Ra and H37Rv infected cells. (E) Gene ontology enrichment analysis on the genes undergoing AS was performed using GOrilla tool [69]. Highly enriched functional classes across each time point were manually classified into broad major functional classes for clarity and plotted above. Metabolism class mostly covered nucleic acid metabolism, whereas genes related to splicing and polyadenylation were covered under RNA processing. Only those classes with more than 10 genes and p-value less than 0.001 were selected for this comparative analysis.

For a gross estimation of alternate splicing events in H37Ra and H37Rv infected macrophages, we compared the junction read counts that originated from exon-exon boundaries across different samples. While the alignment of reads to exon junctions is purely coincidental, we did observe gross differences in the total number of reads corresponding to the exon-exon boundaries between H37Ra and H37Rv infected cells across all the samples (S3B Fig). For a more statistically qualified understanding of alternate splicing in these macrophages, we followed the robust Bayesian analysis framework MATS (Multivariate Analysis of Transcript Splicing) [45]. AS events were classified into five major groups: Alternative 5’ splice site (A5SS), Alternative 3’ splice site (A3SS), Skipped exon (SE), Mutually exclusive exons (MXE) and Retained exon (RI) as reported earlier [45]. In order to get greater insights into the extent of alternate splicing in these macrophages, we calculated “percent splicing index (psi)” score (ψ-score) for each of the transcript variants with respect to the uninfected control samples [20]. A very stringent cut-off for difference of psi-score from uninfected sample (0.5) was taken as significant differential splicing events, which were induced upon infection with H37Ra or H37Rv [20]. In Fig 3A, the number of each of the 5 possible AS events with a psi-score difference of more than 0.5 with respect to uninfected is listed (Fig 3A). The complete list of AS events and corresponding psi-scores across all the samples and time points is provided in S5 Table. Switch like events were detected where the difference in psi-score was exactly 1 or -1. We next plotted ψ-scores of transcripts in H37Ra infected cells (X-axis) versus that of H37Rv infected cells (Y-axis) across the course of infection (Fig 3B). The plots clearly showed that in addition to the AS events being specific to infection with *Mycobacterium tuberculosis*, all data points at the top right in grey in Fig 3B (green box) show significant AS in both H37Ra and H37Rv infection, a large number of them were specific to the infecting strains, indicating differential regulation between H37Ra and H37Rv infected macrophages differing by 0.5 or more (in red and blue respectively, Fig 3B). More switch like events were detected in H37Rv infected samples compared to H37Ra infected samples, which can be easily visualized in Fig 3B as alignment of several red dots on the top left boundary of the psi-score plots at 0, 6, 12 and 36 hours. In H37Ra infected cells, 48 hours sample showed very high number of switch like events (blue dots at bottom, right boundary; Fig 3B). A list comprising genes that show psi-score of 1 in H37Rv infected macrophages is provided in Table 1. While the list in Table 1 does not necessarily captures the whole set of genes, which show AS upon infection with H37Rv, it nevertheless reflects that even for the AS cases as specific as having a psi-score of 1, large number of genes with apparently varying functions are regulated through this means (Table 1). The exact numbers of common and differential splicing events between H37Ra infected and H37Rv infected macrophages across all time points are shown in Fig 3C. Strain-specific splicing patterns got strongly expressed at later time points specifically at 24, 36 and 48 hours post-infection as the total number of genes showing significant splicing and unique to the infection was consistently higher than 1500 at these time points (first two rows combined, Fig 3C). In addition, we also analyzed psi-score distribution for those genes, which were significantly regulated in our analysis in Fig 1. These six additional psi-score plots in supplemental S4 Fig highlight that as we go later in the course of infection, more genes that are differentially regulated at gene level start showing significant alternate splicing as well (Fig 3D and S4 Fig). To understand which biological functions were majorly targeted through AS upon *Mtb* infections, we performed a gene ontology analysis of the genes showing significant alternate splicing unique to either H37Ra or H37Rv infected cells. The large number of functional classes associated with the respective gene list is shown in S6 Table. To infer from the GO analysis, we manually checked for functional classes and classified them into one of the following categories like metabolism, trafficking, redox, gene regulation, cell cycle, lipid metabolism, RNA processing, DNA damage/repair, protein transport, apoptosis, ubiquitination etc. and clubbed the smaller redundant functional classes into large parent functional class. The pi-charts of genes belonging to these functional classes at each of the time points are shown in Fig 3E. Genes belonging to certain classes like metabolism, gene regulation and trafficking or organelle organization undergo alternate splicing throughout the course of infection, irrespective of the infecting strain. Though there were differences in the list of genes within each functional class between H37Ra and H37Rv infection cases (S6 Table). At 48 hours post infection, H37Ra infected cells had more functional perturbations, as evident with more functional classes at 48 hours (Fig 3E). Similarly, at 12, 24 and 36 hours post-infection, H37Rv infected cells showed maximum functional perturbations, as evident by the number of functional classes in the pie-chart (Fig 3E). Some interesting functional classes, which were more perturbed via splicing in H37Rv infected cells include protein transport (12 and 36 hours), redox (36 hours), RNA processing (12, 24 and 36 hours), lipid metabolism (24 hours), translation (36 hours) and ubiquitination (12, 24 and 36 hours; Fig 3E). Together, it was clear that infection induced alternate splicing of transcripts was widespread and therefore had potential to dramatically alter the host response to infection.

**Table 1:** List of genes showing unique switch-like splicing patterns (psi-score of 1) in H37Rv infected macrophages with respect to uninfected control.

**0 Hour**		**6 Hours**	**12 Hours**		**24 Hours**		**36 Hours**		**48 Hours**
ANKRD28	PHF15	ACPL2	APOL3	PRDM10	ADCY7	TMEM107	ABCB7	NARS2	ASIC3
ASPHD1	PKD1	ATP11B	ARHGAP27	RASSF4	APOPT1	TUBB6	ABI2	NDUFB8	ASPSCR1
ATP13A2	PLCB2	BBS1	ARMCX4	RIF1	ATAD3B	UNC5B	AC037459.4	NICN1	ATP11B
ATP2B4	PLCD3	BRF1	ATF7	RINL	BAX	USP54	ACAD8	NME4	ATP5S
AURKA	PLXNB1	BZRAP1	ATP6V0E2	RPRD2	BPTF	VEZT	ADAM15	NR4A1	BAIAP2
BIRC6	PPAPDC1B	C1orf101	ATP9B	SAP30BP	C14orf93	VPS33B	ALKBH2	NUP98	BEAN1
BRD9	PPP1R32	CA13	BIN1	SEMA4F	C19orf40	ZBTB49	ARHGAP11A	OSBPL9	CBWD5
CACNB3	PPP2R5C	CACNA1G	BTBD10	SLC6A12	C9orf116	ZNF23	ATL3	OSCAR	CMC2
CAMTA1	PSTK	CADM1	C19orf44	SMAD7	CCDC146	ZNF268	BCAP29	P4HA1	CNIH3
CARS2	RAD9B	CRB1	C1orf159	SMG1	CCDC64	ZNF280C	C12orf76	PDGFRB	CYP27B1
CCDC66	RAPGEFL1	DMD	CASP9	ST6GAL1	CCNB1IP1	ZNF324B	C16orf62	PFKFB3	DMTF1
CCL4L1	RFX5	DNAJC27	CATSPER1	TAF1A	CDK5RAP3	ZNF565	C6orf223	PHF15	EDNRA
CDKN3	ROBO3	EDNRA	CC2D1A	TAF1D	CDKL3	ZNF691	CAMSAP1	PHF17	EFR3B
CNIH3	RTTN	EIF4G1	CCDC92	TARSL2	CHMP7		CATSPER1	PITPNC1	ENKUR
CNOT7	RUVBL2	EPB41L1	CCNF	TBC1D3	CLN3		CBR4	PKN2	FAM124B
CPSF3L	SEMA4F	FAM131B	CERS4	TDRKH	CMC2		CCNB1	PKN3	FCRL6
CRTC3	SENP6	FAM219B	CNOT8	TIAL1	CPNE1		CD163	POC5	FHAD1
CTD-3203P2.2	SERF2	FAM81A	COPS5	TM7SF2	CTD-2619J13.8		CD1C	PPCDC	GIT2
CUL9	SLC26A1	FSTL3	CYB5R1	TMEM161B	DHRS1		CDC42SE2	PPP3CC	GPR56
CYP4V2	SLC27A1	GLTP	DENND1A	TMEM259	DHX33		CEP250	PSD4	HSPH1
DIP2A	SLC6A12	KCNH2	DGKA	UBR4	DIAPH1		CERKL	PTPN12	LLGL2
DMTF1	SNX19	KCNQ1	DISC1	VARS2	DSTYK		CHIC2	PXN	LRRK1
DNAJC27	SPG21	LRRK1	DONSON	VPS33B	DUSP22		CHURC1-FNTB	RNF4	MECR
DNASE1L1	ST5	LYSMD4	ECT2	WDR45	EIF3L		CSF3	RP1-130H16.18	METTL25
DPCD	ST8SIA5	NBPF3	EXOC6	WDR49	EP400NL		CSF3R	RPS6KA1	MMP8
DYRK4	STAB1	NDUFA4L2	FBXO44	XBP1	EXO1		CTH	RPUSD1	MPHOSPH9
EFCAB11	SYP	NEB	GDPGP1	ZACN	FAAH		CTR9	SCFD1	NBPF3
EIF3E	TARSL2	NPR3	GLS	ZNF12	FAM76B		DCLRE1C	SEH1L	NEB
EP400NL	TDG	PACSIN2	HELQ	ZNF180	FASTK		DDX19A	SERF2	NHSL1
ERMN	THADA	PLCL1	ICAM3	ZNF226	FNIP1		EBPL	SGSH	NRP2
EVL	TKT	POR	IKZF1	ZNF324B	FUK		ENOSF1	SIMC1	OGG1
FAAH	TMEM254	RASGRP4	IL1RN	ZNF528	HDAC9		EPG5	SLC25A29	PAPOLG
FAM76B	TMEM53	RGS18	ILK	ZNF544	KIAA1109		ERMP1	SLC25A43	POR
FBXL8	TONSL	SARM1	INVS	ZNF706	LGALSL		FAM129A	SLC35F6	PTPN18
FBXO36	TOP3A	SDHD	KIAA0040	ZNF821	MAP2K3		FAM179B	SMC4	RPS6KA1
FLYWCH1	UQCRC2	SEP11	LBX2		MUSTN1		FBXO44	SMG7	SDHD
GSG1	VARS2	SPATA7	LIN7B		NAA60		FOSL1	SPAG5	SENP6
HACL1	WDPCP	TFPI	LTBP2		NKAIN1		GINS3	TANGO2	SHISA5
HDAC11	WDR90	TRIM16	MFSD10		NPR3		GON4L	TERF1	SMARCA1
HKR1	XRCC6BP1	UQCC	MLH3		P2RX7		GTPBP10	TMEM201	SNX21
HYI	XRRA1	YTHDF2	MOSPD3		PPA2		HDHD1	TMEM67	SPCS2
IL1RN	ZFP1	ZFAND6	MPZL1		PRPF39		HERPUD1	TNPO2	TAPT1
INCA1	ZNF226	ZMAT1	MRE11A		RAB40C		HSD17B7	TTC7B	TP63
ITFG3	ZNF239	ZNF699	MRPL52		RAD51D		IFT172	TULP3	TXNL1
KCNQ1	ZNF337	ZNF7	MS4A14		RAPGEF2		IL17RA	UBE2T	UCP2
KIRREL2	ZNF385D		MTR		RASSF8		KDM5A	UBL4A	UROS
KRT78	ZNF789		MYBPC3		RNF121		LDLR	UBR4	VPS33B
MAMDC4			NAT6		RSG1		LONP2	UGGT2	ZFAND6
MAMLD1			NDUFA4L2		SCRIB		LRCH3	USP37	ZNF232
METTL21B			NEK3		SKP1		LRMP	WRAP53	ZNF462
MRPS22			NPIP		SLC3A2		LSM2	ZCCHC10	
MS4A6A			PACSIN3		SMARCA1		MAP2K4	ZFAND2B	
MSANTD2			PATL2		SPATA20		MAPK10	ZKSCAN5	
MUSTN1			PHF11		SPATA7		MDM2	ZNF280D	
NFAT5			PHF21A		SPG21		METTL21B	ZNF562	
PACSIN3			PIGB		SSX2IP		MFSD8	ZNF653	
PAPOLG			PLCD3		ST5		MORN2		
PATZ1			PPAT		STRN3		MTERFD2		
PDK1			PPCDC		TAOK3		NAGLU		

### Different transcripts of a gene have several known 3’ UTRs, differing mostly in length

**Fig 4:**
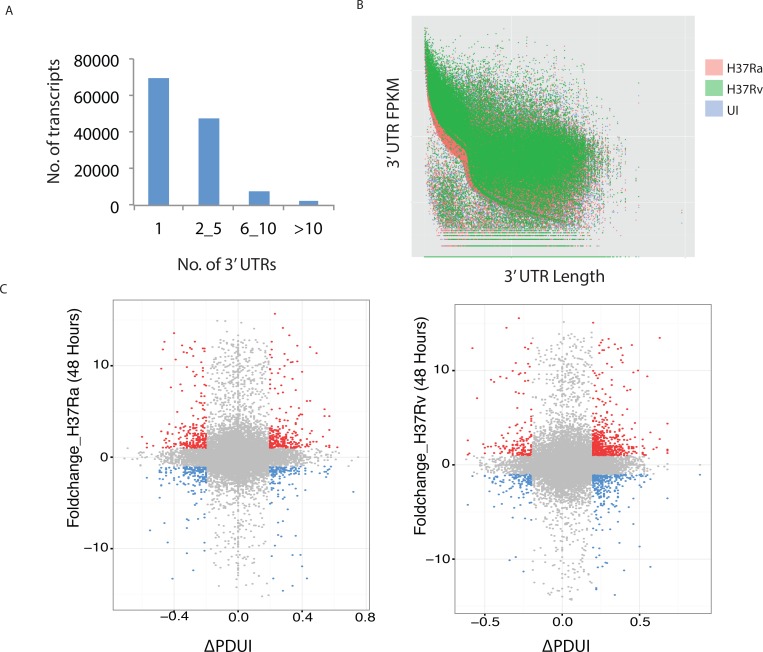
Mtb infection induced alternate polyadenylation in THP-1 macrophages. (A) Number of transcripts in the human genome known to have 1, 2 to 5, 6 to 10 or more than 10 reported 3’UTRs is shown here as obtained from NCBI database. (B) Plot comparing the length of 3’UTR and corresponding FPKMs across the uninfected, H37Ra infected or H37Rv infected macrophages is shown here for 36 hour time point. (C) Percent distal poly-A uses index (PDUI) for transcripts and corresponding FPKM values are plotted here. Transcripts falling below the PDUI threshold score of ±0.2 and corresponding expression level below 2 are shown in grey. Increased expression is highlighted by red and decreased expression by blue.

Various transcripts/isoforms of a given gene vary in the length of 3’ untranslated regions (UTRs). Searching through the Ensembl database revealed even a single transcript could have several different 3’UTR lengths. Fig 4A shows the number of transcripts and their known numbers of 3’UTR as reported in the Ensembl database. A global analysis comparing the 3’UTR length versus the corresponding FPKM revealed that UTR length expression distinctly varied between uninfected and H37Ra or H37Rv infected macrophages (Fig 4B). For a better understanding of the 3’UTR size variation and their respective expression levels, we classified UTRs into size ranges and looked for their expression in terms of FPKM. There were infection and strain-specific variations in the number of transcripts expressing longer or shorter 3’UTR (S5 Fig). The 3’-UTR length may be regulated through the use of proximal or distal poly-adenylation signals (poly-A signal) present in the transcript; a process also termed as alternate poly-adenylation [30]. Higher use of proximal poly-A sites will result in more transcripts with smaller UTR while distal poly-A uses will result in longer UTR. We next calculated percent distal poly-A site uses index (PDUI) score to compare the relative UTR length versus expression using DaPars algorithm [46]. DaPars algorithm has an inbuilt system to notify statistically significant events. The table with PDUI score and corresponding p-values is provided as S7 Table. It turned out; there was a distinct shift in the PDUI score in H37Rv infected macrophages, suggesting an overall increase in the uses of distal poly-A sites and increased expression of transcripts with longer 3’-UTR (Fig 4C). As expected, transcripts with longer UTR showed more differential regulation as compared to transcripts with shorter UTR length (Fig 4C).

In the literature, it is reported that AS and APA events may be linked, suggesting transcripts undergoing AS also have increased chances of undergoing APA [29]. In our data, we could observe nearly 10–15% of transcripts that showed APA also showing AS. These numbers were considerably higher than overall percent significant AS events (0.8–1.6%) and percent significant APA events (0.2–2.6%) alone thereby suggesting AS and APA to some extent indeed may be linked events. Gene ontology enrichment of the list present in S7 Table showed genes from a majority of functional classes undergoing APA including those involved in splicing, phagocytosis, immunity, apoptosis and metabolism (S8 Table) again emphasizing the global APA events as a consequence of *Mtb* infection.

### Extensive alternate splicing of the components of the spliceosome complex

**Fig 5:**
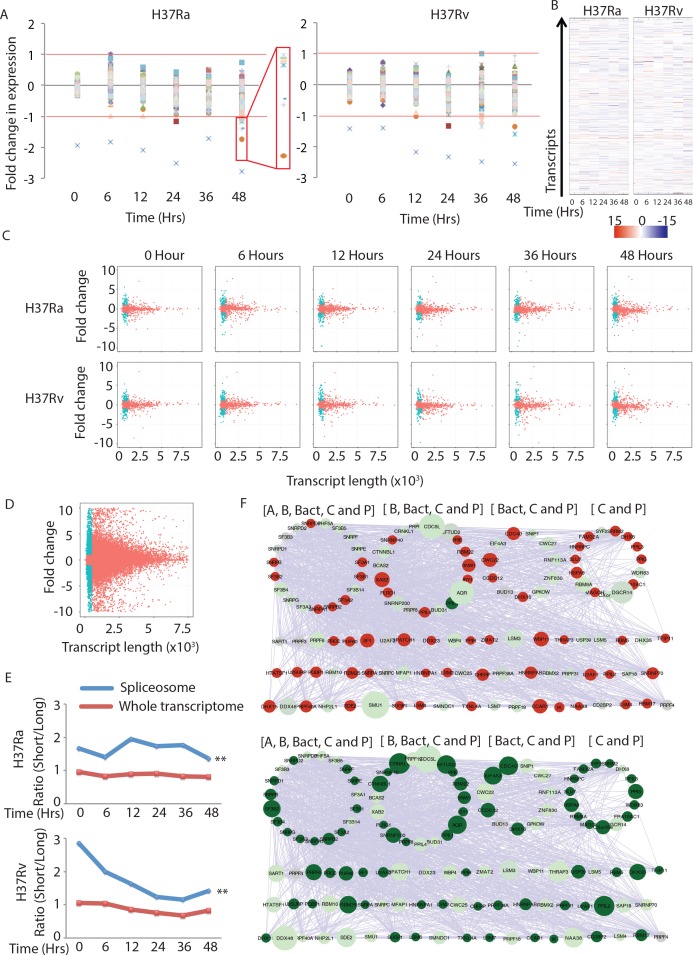
Regulation of spliceosome machinery upon infection with *Mtb*. (A) Expression level (fold change with respect to uninfected control) of genes from the list obtained from the spliceosome database. The red line marks the threshold for regulation i.e. +1 (two fold increase) and -1 (two fold decrease). The blue cross marker identifies *PRPF19*, which is significantly down regulated across each time points in both H37Ra and H37Rv infected cells. The red box in H37Ra, 48 hours has been zoomed to identify molecules at the right. Starting from the top these molecules are: *SNRPF*, *RBM8A*, *SNRPC*, *PPIL1*, *NOSIP*, *SNRPB* and *LENG1*. The markers for molecules are same in both the plots. (B) For every gene identified in the spliceosome database, corresponding expression values for all known transcript variants was selected from the transcript expression table (S9 Table) and plotted as the heat map here. Each column corresponds to the time point as shown. The pseudo color bar below the plot explains the fold change values at transcript level in the heat map. (C) Plots show length versus fold change in expression for each of the transcripts of the spliceosome genes under mentioned conditions. The dots in blue represent transcripts less than 1000bp and with the expression value of more than 1 or less than -1. (D) Similar plot as in 5C for the entire transcriptome for one of the time point (H37Ra, 48 hours). The dots in blue represent transcripts less than 1000bp and with the expression value of more than 1 or less than -1. (E) From the transcripts list representing spliceosome genes and for the entire transcriptome, number of transcripts less than or more than 1000bp size with significant regulation was calculated. The ratio of number of shorter regulated transcripts to that of regulated longer transcripts are plotted here for H37Ra and H37Rv infected macrophages across the time points. Blue line represents ratio for the spliceosome related transcripts while red line represents ratio for the entire transcriptome at the respective time points (** p-value <0.0001 across each of the time points in both H37Ra and H37Rv infected conditions). (F) List of genes belonging to different stages in the spliceosome assembly and activity (see text) were queried in STRING database for functional associations. The interaction network thus built was then organized in a manner that genes that were shared at multiple stages were clubbed together in a circle, while retaining the interactions with other components. Thus, molecules in the top left circle were present at each of the five major stages of spliceosome assembly/actions: A, B, Bact, C and P (as marked). Subsequently, size of each node was defined based on the length of the transcript that get maximally up regulated (in the top panel) or maximally down regulated for each of the genes (lower panel). Red and green colors in both top and lower panel identifies fold change in expression values more than 1 and less than -1 respectively. The expression data used to color and size the nodes here was from H37Rv infected cells at 48 hours post-infection.

Having witnessed considerable changes in the splicing pattern in macrophages post-infection, we were curious to test whether genes belonging to the different stages of spliceosome complex could also undergo alternate splicing. We downloaded the list of genes involved in splicing events from the spliceosome database [47]. Off nearly 131 genes involved in splicing, a vast majority did not show any significant regulation in either virulent or avirulent infection at any of the time points studied (Fig 5A). Among few that were differentially regulated, PRPF19 was significantly down regulated across all the time points in both H37Rv and H37Ra infected macrophages (Fig 5A). Few more genes, which showed down regulation at 48 hours in H37Ra infected cells, included LENG1, NOSIP, PPIL1, RBM8A, SNRPB, SNRPC and SNRPF. Out of these LENG1 and PPIL1 were also down regulated in H37Rv infected macrophages at 48 hours post-infection (Fig 5A). We next compared the expression of the spliceosome-associated genes at transcript levels. At the transcript level the scenario was grossly different with a large number of transcripts showing marked regulation in expression with respect to the control in both H37Ra and H37Rv infected macrophages (Fig 5B). The list of transcripts associated with spliceosome genes and their corresponding expression level at each of the time points is provided in S9 Table. The range of transcript expression varied between ~15fold down regulation to ~12 fold up regulation (Fig 5B). While alternate splicing may result in varied lengths of the final product, we were especially intrigued with the frequency of shorter or truncated transcripts, almost universally present for most of the genes. These transcripts usually spanned lesser than 1000bps and multiple variants of these transcripts were present for a large number of genes. Shorter transcript variants in many instances do not get translated or when translated, do not form a functional protein. Transcripts with premature stop codon get degraded through nonsense-mediated decay (NMD;[48, 49]. To test how the expression of these truncated transcripts was regulated, we plotted transcript length versus corresponding fold change in expression for each of the transcripts of genes associated with spliceosomes across all the conditions (Fig 5C). A cursory examination of the plots revealed that more number of transcripts shorter than 1000bp was differentially regulated than those transcripts that were longer than 1000bp. As shown in Fig 5C, for each of the condition/time-points the plots clearly reveal this bias. For comparison when we plotted the entire transcriptome data for any condition, this bias was visibly lost or at least declined (Fig 5D). To get a numerical representation of this bias in the relative enrichment of transcript length among highly regulated transcripts, we calculated the ratio of the number of shorter transcripts differentially regulated to that of the number of longer transcripts differentially regulated for each experimental groups. This ratio was calculated separately for transcripts associated with spliceosome genes or for the entire transcriptome (Fig 5E). The plots in Fig 5E show that among the spliceosome genes there is a far greater propensity for the enrichment of differentially regulated shorter transcripts compared to the whole transcriptome. The differences were also statistically significant as we confirmed through a hyper-geometric analysis (Fig 5E). It was true for each of the time points studied in both H37Ra and H37Rv infected macrophages (Fig 5E).

Finally, we captured the transcript variants and their relative expression on a protein functional association network between the genes involved at different stages of splicing like complex A, complex B, complex Bact, complex C and complex P [28]. For all the proteins known to be part of the spliceosome complexes as published earlier (28) their interaction pattern was obtained from STRING database to create a functional association network of spliceosome components. Since different stages of spliceosome assembly involve several common and unique molecules, we partitioned the network into smaller circular sub-clusters, each of them common to a set of splicing complexes (Fig 5F). Thus, the first circle on the top, left of the network included molecules that were part of each of the five spliceosome stages A, B, Bact, C and P (Fig 5F). Similarly, other circles were created specifying the spliceosome stages they represented. Following that, the expression value (either maximum or minimum, represented by colors red or green respectively) and length of the corresponding transcript (represented by the size of the nodes) were incorporated into the network to visualize how the spliceosome machinery was perturbed upon infection. We used expression data at 48 hours post-H37Rv infected macrophages for this analysis. As is visually evident from Fig 5F, more transcripts of shorter length were up or down regulated at multiple steps of the spliceosome function (Fig 5F). This analysis corroborated with the earlier observation that shorter transcripts corresponding to the spliceosome related genes were more differentially regulated as compared to their longer counterparts. Together Fig 5 highlights that the observed variations in the pattern of splicing in infected macrophages influence the spliceosome components.

### Selective abundance of truncated, non-translated transcripts of key genes in H37Rv infected macrophages

**Fig 6:**
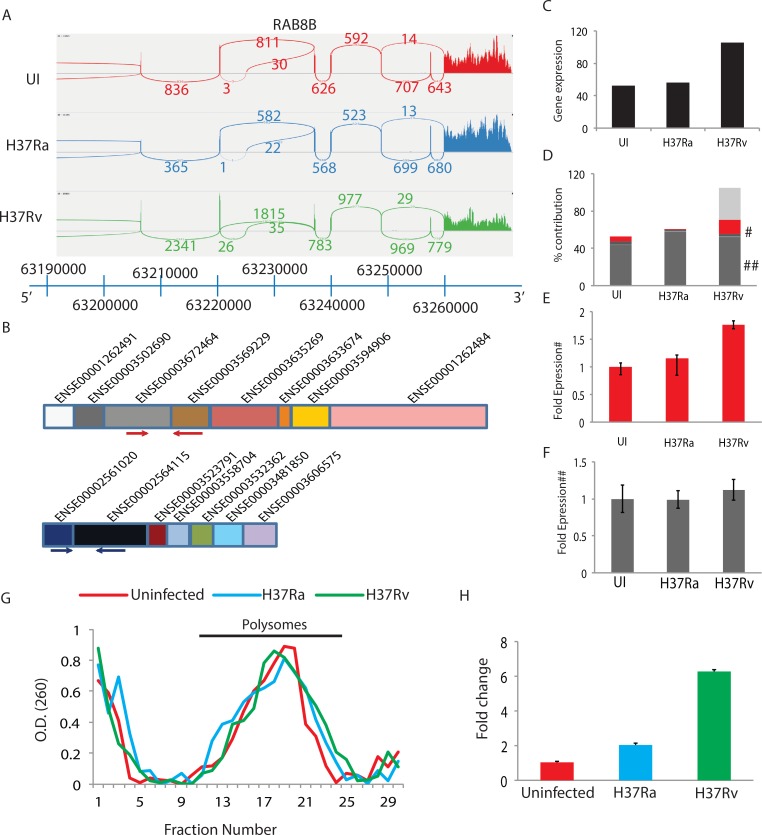
Enrichment of truncated transcripts due to AS. (A) SASHIMI plots for reads alignment at *RAB8B* locus. Number of reads corresponding to specific exon-exon junctions (shown as loops) is labeled for each junction. Only partial *RAB8B* locus is shown here for clarity. (B) The exon organizations of RAB8B isoforms are shown here. Each colored box corresponds to a unique exon (identified by the unique exon ID). The red and blue arrows mark the primer sites picked-up to validate through Q-PCR, longer and shorter transcripts respectively. (C) Gene level quantification of RAB8B expression in UI, H37Ra infected or H37Rv infected THP-1 macrophages as observed in RNA-seq data. (D) Percent contribution of RAB8B transcripts across the three groups, as observed in RNA-seq data. The red bar corresponds to the shorter transcript analyzed in this study. Marks of ^#^ and ^##^ correspond to shorter and longer transcripts respectively in the plot, which were subsequently validated through Q-PCR. (E) Fold expression of RAB8B shorter transcript validated by Q-PCR in an independent set of experiments. (F) Fold expression of RAB8B longer transcript validated by Q-PCR in an independent set of experiments. (G) Isolation of polysome fraction using density gradient centrifugation from the uninfected, H37Ra infected or H37Rv infected THP-1 macrophages at 48 hours post-infection (see methods for detail). (H) Q-PCR for RAB8B shorter transcript in the total RNA isolated from the polysome fraction.

As seen above, infection with *Mtb* led to an increase in the expression of truncated transcripts for splicing related genes. Functional analysis of highly spliced variants too revealed several genes belonging to key functional classes like immune regulation and response to stress getting enriched in the list of alternate spliced genes. We decided to investigate the influence of alternate splicing particularly that of truncated transcript on shaping the cellular response to infection. We verified the increased expression of shorter transcripts in the infected macrophages by performing isoform-specific real-time PCR analysis of genes like *RAB8B*, *ACSL1* and Dynamin-1 (Fig 6 and S6 Fig). Selection of these genes for subsequent analysis was with keeping in mind their known association with the aspects of trafficking (RAB8B and Dynamin-1) as well as lipid metabolism (ACSL1), both key functional aspects in the virulence [42, 44]. To visualize the extent of alternate splicing, we constructed SASHIMI plots for *RAB8B*, *ACSL1* and Dynamin-1 (Fig 6 and S6 Fig). SASHIMI plots integrate the probability of a splicing event keeping into account reads corresponding to the exon-exon junctions [50]. As shown in Fig 6 and S6 Fig, in each of the three cases analyzed, considerable differences were observed in terms of the number of reads corresponding to exon-exon junctions between uninfected or H37Ra or H37Rv infected macrophages. The comparative exon organization plots for the shorter transcript with respect to the full-length transcripts are shown in Fig 6B and S6 Fig). The shorter transcript of *RAB8B* (ENST00000558990) had completely different exon composition with respect to the full length *RAB8B* (ENST00000321437) transcript (Fig 6B). At the gene level, expression of *RAB8B*, *ACSL1* and Dynamin-1 was minimum in case of uninfected macrophages, intermediate in case of H37Ra infection and highest in the case of H37Rv infection (Fig 6C and S6 Fig). We then calculated percent contribution of each of the known transcripts of *RAB8B*, *ACSL1* and Dynamin-1 (Fig 6D and S6 Fig). Surprisingly, a large proportion of the increase in expression witnessed in H37Rv infected macrophages at the gene level was contributed by an increase in the expression of the corresponding truncated transcripts in *RAB8B* and *ACSL1* (Fig 6D and S6 Fig). In the case of Dynamin-1, even H37Ra infected macrophages showed increased expression of the truncated transcript (S6 Fig). Using isoform-specific primers, designed from independent exon-exon boundaries, we then validated through real-time PCR, increased expression of the truncated isoforms of *RAB8B* and *ACSL1* (Fig 6E and S6 Fig). For real-time experiments, we used both β-Actin and 18srRNA as controls in independent experiments. Expectedly, as seen in the case of RNAseq data, there was no difference in the expression of longer transcript between UI, H37Ra or H37Rv cells (Fig 6F). We were keen to understand whether and how increased expression of the truncated isoforms could influence cellular responses to infections. The truncated *RAB8B* transcript ENST00000558990 does not get translated since it has a premature stop codon. It rather undergoes nonsense-mediated decay (NMD), a process that keeps quality control of mRNAs [51]. NMD takes place while the mRNA is still part of the translational machinery. We, therefore, isolated the polysome fraction from uninfected, H37Ra infected and H37Rv infected THP-1 macrophages, (Fig 6G). From the total RNA isolated from the polysome fraction, we checked for the presence of truncated transcripts. In the case of RAB8B, we could observe a nearly six-fold increase in the level of truncated transcript in the polysome fraction from H37Rv infected macrophages as against the uninfected control (Fig 6H). Even in the case of H37Ra infection, there was nearly two-fold increase in the enrichment of truncated isoform in the polysomal fraction.

### Relative levels of transcript variants influence cellular responses to *Mycobacterium tuberculosis* infections

**Fig 7:**
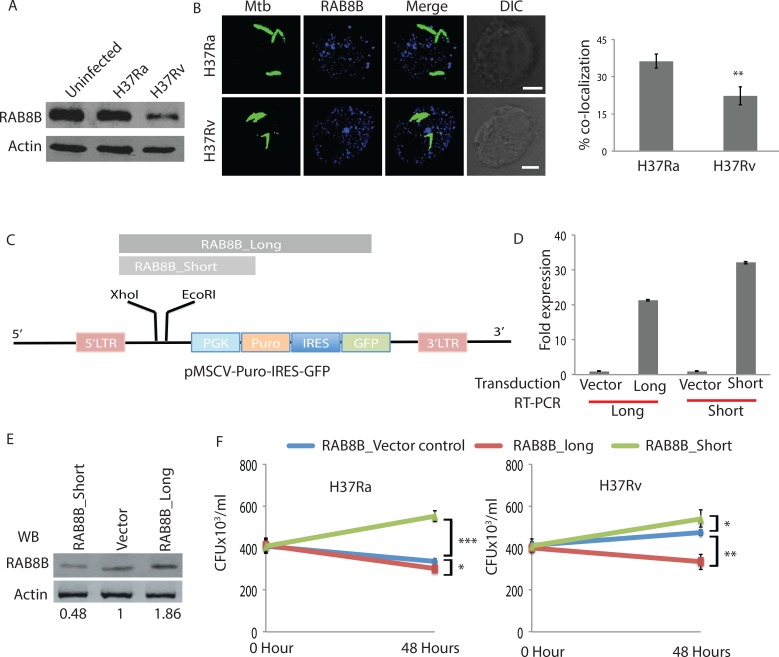
Regulation of intracellular *Mtb* survival by longer and shorter isoforms of RAB8B. (A) RAB8B immunoblot from the lysates of THP-1 macrophages infected with H37Ra, H37Rv or uninfected at 48 hours time point. Actin was used as the loading control. (B) THP-1 macrophages were infected with GFP-expressing H37Ra or H37Rv (green) at MOI of 1:10. At 48 hors post-infection, samples were fixed and immunostained with anti-RAB8B antibody (red). Representative confocal microscopy images are shown in the left. Number of *Mtb* phagosomes showing co-localization with RAB8B was manually counted using Imaris 7.2 image analysis software. Data represents average of more than 60 phagosomes from at least 10 fields from three different replicates (values: mean±SEM, **p-value<0.001; scale bar: 5μm). (C) Long and short transcripts of RAB8B were cloned in the pMSCV-PIG lentiviral vector. GFP is preceded by an IRES element downstream to puromycin selection marker under PGK promoter. (D) From THP-1 macrophages transduced with vector control, RAB8B_long or RAB8B_short lentiviruses; cDNAs were prepared. Relative expression of RAB8B long or short transcripts was confirmed by RT-PCR using specific primers. (E) THP-1 macrophages transduced with vector control, RAB8B_long or RAB8B_short lentiviruses were lysed at 48 hours post-transduction and probed for RAB8B by Western blot. Numbers show densitometric quantification normalized to Actin. (F) THP-1 macrophages transduced with vector control, RAB8B_long or RAB8B_short lentiviruses were infected with H37Ra or H37Rv. At 0 hour (see methods) and 48 hours post-infection, cells were lysed and number of surviving bacteria was counted by plating on 7H11 plates. At both 0 hour and 48 hours, samples were also analyzed through FACS to ascertain percent THP-1 cell population positive for GFP (S7 Fig).

We next verified the effect of alternate splicing on RAB8B protein levels and whether that influenced host response to *Mtb* infections. Estimation of relative protein abundance of RAB8B revealed that increased expression of the truncated transcript correlated with a sharp decline in the protein levels in H37Rv infected macrophages (Fig 7A). It was true for ACSL1 proteins as well (S7A Fig). How increased expression of truncated transcript led to a decline in RAB8B protein level remains less understood. However, one possibility is that it can compete with the full-length variants for translational machinery. RAB8B belongs to the family of small monomeric GTPases, involved in the process of intracellular trafficking [44, 52]. This protein also seems to influence the maturation of *Mtb*-containing phagosomes and autophagosomes [44]. We compared recruitment of RAB8B to GFP-expressing H37Ra and H37Rv phagosomes in THP-1 macrophages at 48 hours post-infection. While H37Ra showed nearly 36% co-localization with RAB8B, it was significantly lower (22%) in the case of H37Rv infections (Fig 7B). H37Ra is known to get readily targeted to lysosomes unlike H37Rv [53]. It, therefore, seemed plausible to hypothesize that by decreasing the RAB8B protein levels through this unusual means of post-transcriptional regulation, the virulent strain of *Mtb* evades getting targeted to the lysosomes and therefore promotes its survival. To further test this hypothesis we cloned the full-length or the truncated transcript cDNAs of *RAB8B* into the pMSCV-PIG vector where the expression cassette contains GFP as part of a bicistronic construct separated by an IRES element (Fig 7C). Lentiviruses were made by transfecting these constructs into HEK293T cells along with the helper plasmids and then transduced into THP-1 macrophages. The purpose of this experiment was to test whether by overexpressing shorter or longer transcripts of *RAB8B*, survival of *Mtb* could be regulated within THP-1 macrophages. We were able to achieve more than 70% transduction of long and short *RAB8B* transcripts as well as vector controls in THP-1 macrophages at the time of infection as well as 48 hours post-infection (S7B Fig). The real-time analysis confirmed increased expression of both shorter and longer transcripts upon transduction (Fig 7D). Western blot analysis of transduced THP-1 macrophages, interestingly, correlated with the pattern observed above in H37Rv infected macrophages. Thus, whereas cells transduced with longer *RAB8B* transcript showed a higher level of RAB8B proteins, those transduced with shorter *RAB8B* transcript showed a diminished level of RAB8B proteins with respect to vector control (Fig 7E). We next infected these macrophages with H37Ra or H37Rv and monitored their survival. In vector control transduced cells, H37Ra and H37Rv survival followed the expected pattern, resulting in higher H37Rv CFU at 48 hours post infection compared to H37Ra (Fig 7F). There was no difference in the uptake of bacteria in THP-1 macrophages under any of the three conditions (Fig 7F). In cells transduced with shorter *RAB8B* transcript, both H37Ra and H37Rv showed higher CFU compared to vector control (Fig 7F). Similarly overexpressing longer *RAB8B* isoform led to diminished CFU for both strains with respect to the vector control (Fig 7F). It is important to note here that the effect of overexpressing longer transcript on H37Ra survival (decline of nearly 11% with respect to vector control) and that of shorter transcript on H37Rv survival (increase in CFU by ~15%) were although significant but of lower magnitude than vice versa i.e. ~60% increase in H37Ra CFU in cells transduced with shorter transcript while ~30% decline in H37Rv CFU in cells transduced with longer transcript (Fig 7F). It possibly reflected already high RAB8B protein levels in H37Ra infected cells and low RAB8B protein levels in H37Rv infected cells. In fact, H37Rv infected macrophages that were also transduced with shorter *RAB8B* transcript showed further diminished RAB8B protein level with respect to H37Rv infected cells alone, thereby confirming the same (S7C Fig). It was now evident, by lowering RAB8B protein level via the unusual means of post-transcriptional regulation virulent *Mtb* strain was aiding its survival within macrophages.

### *RAB8B* is required for phagosome maturation and killing of *Mtb*-infected macrophages

**Fig 8:**
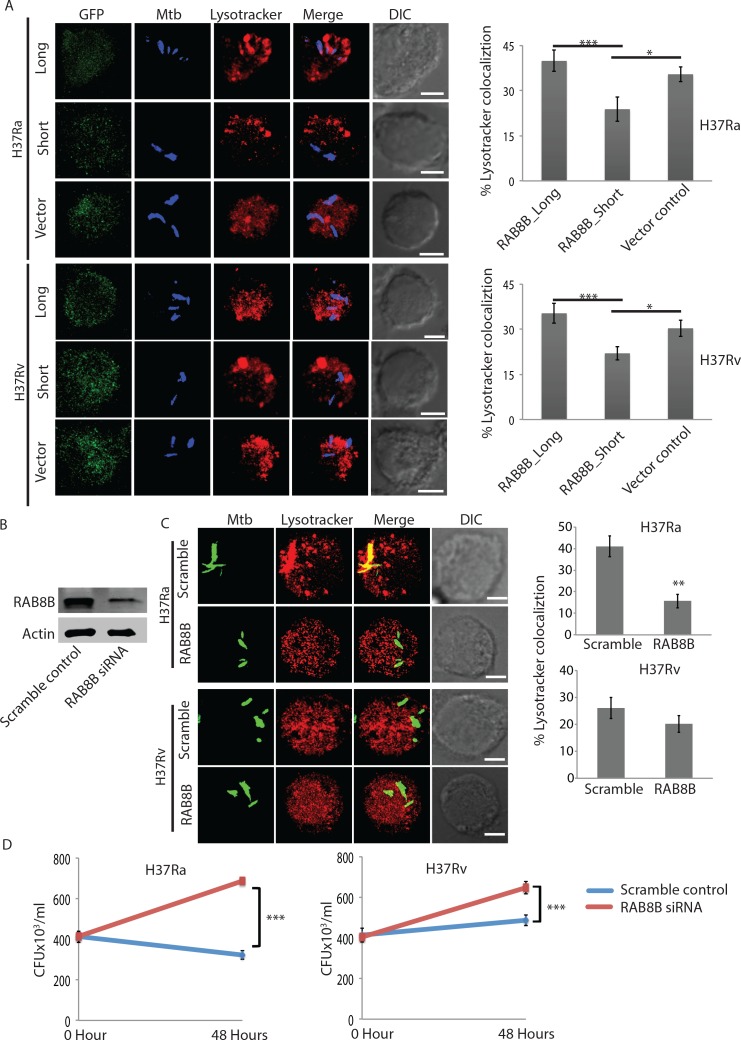
RAB8B regulates intra-macrophage *Mtb* survival by impacting phagosome maturation step. (A) THP-1 macrophages transduced with vector control, *RAB8B*_long or *RAB8B*_short lentiviruses were infected with APC-Cy7 labeled H37Ra or H37Rv (green). At 48 hours post-infection samples were stained with LysoTracker and images were acquired using confocal microscopy. Co-localization of H37Ra and H37Rv to Lysotracker compartment was calculated using Imaris 7.2 software under each of the conditions mentioned. The data in the bar-plots (right) represent average of more than 60 phagosomes from at least 10 fields from three replicates (Values ± SD); *p-value<0.05, **p-value<0.01, scale bar: 5μm. (B) THP-1 macrophages were transfected with either scrambled or *RAB8B* specific siRNA (50nM) and cells were lysed at 48 hours and probed for RAB8B protein levels by Western blot. (C) THP-1 macrophages were infected with GFP expressing H37Ra or H37Rv. Subsequently, *RAB8B* siRNA (50nM) was transfected (see methods) and at 48 hours post-infection samples were stained with Lysotracker for analysis under confocal microscope. Co-localization of H37Ra and H37Rv to Lysotracker compartment was calculated using Imaris 7.2 software under each of the conditions mentioned. The data in the bar-plots (right) represent average of more than 60 phagosomes from at least 10 fields from three replicates (Values ± SD); NS: non-significant, *p-value<0.05, **p-value<0.01, scale bar: 5μm. (D) THP-1 macrophages were infected with H37Ra or H37Rv followed by treatment with scrambled or *RAB8B* specific siRNA (50nM). At 0 hours and 48 hours post-infection (see methods), cells were lysed and surviving bacteria were plated on 7H11 plates for CFU counting. Data represents average of multiple replicates, values±SD.

To understand how RAB8B protein levels influenced *Mtb* survival within macrophages, we analyzed lysosomal targeting of *Mtb* in cells that were transduced with the long or short isoform of *RAB8B* as a bi-cistronic construct with GFP. Expression of GFP allowed us to analyze only those cells, which were transduced. We found levels of RAB8B protein correlated with the lysosomal targeting of *Mtb*. Thus more H37Rv localized to lysosome when cells were transduced with *RAB8B* long transcript compared to vector control (Fig 8A). Similarly lesser H37Ra localized to lysosomes when transduced with short *RAB8B* transcript compared to vector control (Fig 8A). Due to the large variation observed in the confocal microscopy data, mostly due to differential level of expression of the transduced copy of RAB8B, we could not get significant differences between vector control and long isoform group although they followed the expected trend. Transduction of shorter transcript, however, showed consistently significant difference from both vector control and long isoform (Fig 8A). Thus, levels of RAB8B protein influenced phagosomal maturation and survival of *Mtb* within infected macrophages. The levels of RAB8B protein itself, as shown above, was regulated by the pattern of alternate splicing.

To further confirm the specific role of RAB8B in regulating *Mtb* killing, we knocked down *RAB8B* (using siRNA specific to the longer isoform) in THP-1 macrophages that were infected with either H37Ra or H37Rv. We observed nearly 80% knockdown of RAB8B at protein level (Fig 8B). RAB8B knockdown led to decreased targeting of both H37Ra (~60%) and H37Rv (~20%) to lysosomal compartments as compared to scramble siRNA treated control (Fig 8C). Finally, we were also able to observe a significant increase in the survival of both H37Ra and H37Rv in RAB8B knockdown cells (Fig 8D). In agreement with the findings in the previous section, increase in bacterial CFU upon *RAB8B* knockdown with respect to control was more pronounced in the case of H37Ra (~65%) than that of H37Rv (~50%, Fig 8D).

### *Mycobacterium tuberculosis* infection induces alternate splicing of *RAB8B* transcripts in primary human macrophages and impacts pathogen survival

**Fig 9:**
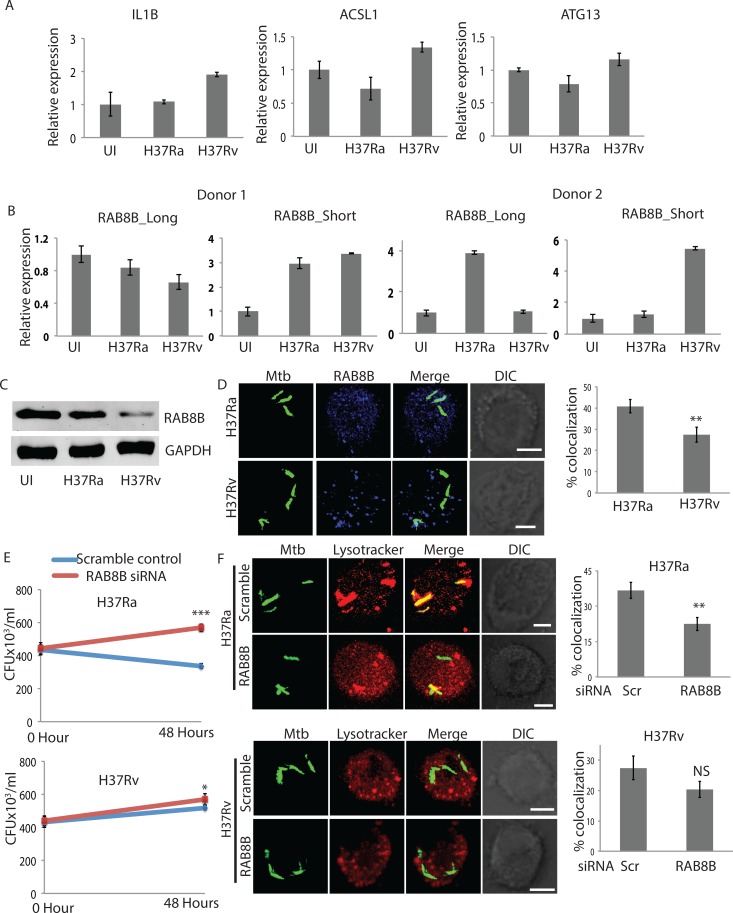
*Mtb* infection induces alternate splicing in human primary macrophages (MDMs) and impacts cellular response. (A) MDMs were infected with H37Ra or H37Rv. At 48 hours post-infection, total RNA was isolated and transcript-specific RT-PCR performed for IL1B, ACSL1 and ATG13. Data was normalized against 18s rRNA expression, values±SD. (B) MDMs from two different donors were infected with H37Ra or H37Rv, expression of RAB8B shorter transcript upon infection analyzed using RT-PCR. Data was normalized against 18s rRNA expression, values±SD. (C) MDMs infected with H37Ra or H37Rv were lysed at 48 hours post-infection and probed for RAB8B protein by Western blot. Numbers are densitometric quantification of blots, representative from two independent experiments. (D) MDMs were infected with GFP expressing H37Ra or H37Rv. At 48 hours post-infection samples were stained with RAB8B and images were acquired under confocal microscope. Co-localization between GFP-Mtb and RAB8B was calculated using Imaris 7.2 software. Plots at the right represent data from more than 60 phagosomes from three different replicates, Values±SEM, *p-value<0.01, scale bar: 5μm. (E) MDMs were infected with H37Ra or H37Rv at MOI 1:10. Subsequently cells were treated with RAB8B siRNA (50nM) and at 48 hours post-infection, cells were lysed and surviving bacteria were plated on 7H11 plates for CFU counting. Data represents average of multiple replicates, values±SD. (F) MDMs were infected with GFP expressing H37Ra or H37Rv. Subsequently, RAB8B siRNA (50nM) was transfected and at 48 hours samples were stained with Lysotracker for analysis under confocal microscope. Co-localization of H37Ra and H37Rv to Lysotracker compartment was calculated using Imaris 7.2 software under each of the conditions mentioned. The data in the bar-plots (right) represent average of more than 60 phagosomes from at least 10 fields from three replicates (Values ± SD); NS: non-significant, *p-value<0.05, **p-value<0.01, scale bar: 5μm.

As noted earlier, alternate patterns of splicing and poly-adenylation has been shown to be associated with different diseases including cancers [54, 55]. It was, therefore, important to test that truncation of RAB8B transcript upon H37Rv infection was not a consequence of the transformed phenotype of THP-1 cells, a leukemic cell line, rather represented a true macrophage response upon bacterial infection. We tested some of the genes analyzed in Fig 2 from monocyte-derived macrophages (MDMs) obtained from PBMCs of healthy volunteers. The pattern of splicing of genes *IL1B*, *ACSL1* and *ATG13* upon infection with H37Ra or H37Rv followed the trend observed in THP-1 macrophages, showing maximum expression in H37Rv infected cells than others (Fig 9A). For *RAB8B*, we confirmed its splicing patterns from two different donors. While both the donors show a similar increase in *RAB8B* truncated transcripts upon H37Rv infection, one of them also showed an increase in truncated *RAB8B* upon H37Ra infection, however levels in H37Ra infected MDMs were always lower than those in H37Rv infected ones (Fig 9B). In fact, in the second donor, longer RAB8B transcript increased manifold upon H37Ra infection, suggesting strong phagosome maturation flux in that individual (Fig 9B). All subsequent validations were done using MDMs from donor 1. We next confirmed, increase in *RAB8B* shorter transcript expression correlated with a decline in RAB8B protein level in MDMs upon H37Rv infection (Fig 9C). In agreement with the results in THP1 macrophages, more H37Ra (~40%) localized to the RAB8B compartment in the MDMs compared to H37Rv (less than 30%, Fig 9D). We were also able to knockdown RAB8B longer isoform in MDMs using siRNAs (S7D Fig). Upon siRNA-mediated knockdown of *RAB8B* in the MDMs, both H37Ra and H37Rv showed an increase in CFU (Fig 9E). Expectedly the increase was more pronounced and significant in the case of H37Ra infection as well as reduced co-localization to lysosomes (Fig 9E). Similarly, upon RAB8B knockdown bacterial co-localization to lysosome declined, which was again more pronounced and significant in the case of H37Ra infection compared to H37Rv infection (Fig 9F). Thus infection induced alternate splicing of *RAB8B* is a specific response of macrophage, which helps the survival of virulent strain in the infected macrophages.

## Discussion

Alternate processing of pre-messenger RNA results in the production of several variants of mRNA of a given gene [20]. Alternate splicing is attributed to play a crucial role in the generation of biological complexity, and arguably, therefore, mis-regulation in the alternate splicing machinery is associated with several human diseases [54]. In fact, it is estimated that nearly 15–50% of human disease mutations directly influence the splice site selection [54, 55].

The role of alternate splicing in the regulation of cellular response, especially in the immune responses, is steadily getting evident. In innate immune regulation, the role of alternate splicing has been extensively studied in the context of TLR4 signaling [22]. The converging theme across several studies in this context was how alternately spliced variants of a transcript result in non-functional and/or truncated variations of the protein, resulting in switching off of the immune signaling [23]. Infection with bacterial pathogens including Mycobacterium is known to induce a sharp change in the macrophage gene expression profile [5, 14]. However, most such studies involved microarray analysis and relied on gene level interpretations. Here we performed RNA-seq analysis and comprehensively investigated transcript variants that were regulated upon infection in macrophages. At gene level classification, functional enrichment analysis did reveal similar functional classes in our study, as reported earlier, like metabolism, immune regulation or inflammation, etc. [5].

Eukaryotic gene expression is a complex process, where primary transcripts are processed at multiple steps including capping, cleavage and polyadenylation, cytosolic export, splicing, editing, etc. [56]. Most of these steps are regulated and could significantly impact the overall stoichiometry and functioning of corresponding translated products [56]. A comparative analysis of gene-level and isoform level expression of several genes showed discordance between them for most of the genes. Thus certain isoforms of a given gene were more enriched in expression and that the identity of enriched isoforms varied between H37Ra infected, H37Rv infected and uninfected macrophages. It strongly indicated that infection with *Mtb* was altering the global patterns of alternate splicing in the macrophages. The psi-score analysis, which factors in the frequency of exons spliced in among different isoforms of a given gene, indeed confirmed infection and strain-specific regulation of alternate splicing. To the best of our knowledge, infection induced alternate splicing at the global level has never been shown in the past. While the altered pattern of splicing itself was novel observation, the functional consequences of it became more evident as we noted for several genes, the truncated isoforms were highly regulated in the infected macrophages. Many of the truncated transcripts are known to have pre-mature stop codons, and they do not get translated [57]. These transcripts are subsequently degraded through a process called non-sense mediated decay [57]. How increased abundance of truncated transcripts influence cellular response is not exactly clear, however in one such study in an unrelated system, it was shown that non-sense mediated decay cramps the nuclear cap binding protein (NCBP) in an inactive form thereby influencing the cytosolic export of newly formed transcripts [58]. It will be interesting to see whether similar mechanisms are operational in higher eukaryotes as well. It is important to note here that we used a highly stringent cut-off (0.5) to select AS events. In the literature, a cut-off of 0.2 is considered significant to identify AS events. At that cut-off, the number of genes showing AS will considerably increase. Thus AS as a consequence of *Mtb* infection may be even more wide-spread than being presented in this study. The global nature of alternate splicing triggered by *Mtb* infection was also evident by our analysis that revealed extensive alternate splicing of genes belonging to spliceosome complex. Since many AS events depend on recognition/non-recognition of weak splice sites [59], loss of specific functional domains of a protein or decrease in the concentration of certain spliceosomal protein due to AS could influence the overall splice site selection and therefore AS. Moreover, it is understood that many important binary interactions between spliceosome complex proteins are weak in nature and require several additional factors to get stabilized [60]. Thus alternate spliced products of spliceosome proteins are likely to vary in the stabilizing properties, further favoring extensive alternate splicing. Given the multiplicity of factors involved, understanding the exact mechanism of AS regulation upon *Mtb* infection would require a more focused study.

We were also intrigued with yet another form of transcript length regulation via alternate polyadenylation [30]. The length of 3’-UTR of transcripts can influence the stability of the transcripts via exclusion/inclusion of miRNA binding sites which typically target the 3’UTRs [30]. Such regulation during transformation has been reported where several genes involved in cell cycle/survival were shown to have a shorter 3’UTR and longer stability resulting in highly efficient translation. Regulation of alternate polyadenylation upon infection has never been discussed in the past. We identified several APA events that were specific to virulent infections. In general, virulent infections resulted in more elongation of transcripts (increased use of a distal poly-A site). That could be another means to destabilize the transcripts and eventually influence the corresponding protein levels in the cells.

Our studies with RAB8B isoforms in regulating cellular responses to infection were quite revealing. While there were several molecules of interest in the AS list, we followed the effect of AS on RAB8B for two reasons. Firstly, RAB8B is known to participate in the phagosome maturation pathway involving RAB7 and TBK1 [44, 52]. Secondly, it showed an interesting pattern where increased expression of truncated transcripts in virulent infection led to a decline in the protein level. Phagosome maturation arrest is an established mechanism through which virulent strains of *Mtb* are known to evade lysosomal targeting [53, 61]. The role of small GTPases like RAB7 is established in the maturation process, and exclusion of RAB7 from *Mtb* phagosomes is one of the core mechanisms helping the bacteria to evade lysosomal targeting [61]. This study adds another dimension to the regulation of phagosome maturation in *Mtb*-infected macrophages, whereby levels of RAB8B, a molecule closely associated with RAB7, gets significantly depleted as a consequence of alternate splicing. That the RAB8B protein levels could be regulated by the relative expression of shorter and longer isoforms was confirmed using transduction studies as well as knockdown studies. Having identified and established the AS-mediated regulation of macrophage response in THP-1 macrophage, which are monocytic leukemia cells, it was critical to test whether similar mechanisms are operational in primary macrophages as well. That is partly because genetic variations in the splice-site are linked to several diseases in humans including cancer [62, 63]. In macrophages derived from peripheral blood of healthy donors (MDMs), we could confirm infection specific alterations in the expression of *IL1B*, *ACSL1*, *ATG13* and *RAB8B* isoforms as noted in THP-1 macrophages. We further verified that selective *RAB8B* splicing influenced the outcome of infection, confirming it to be a true macrophage response. Even with a very limited analysis of RAB8B AS upon H37Rv infection in macrophages from two different donors, it was evident that there is an enormous possibility of inter-individual variations in the patterns of splicing. Therefore it is likely that depending on the extent of AS and APA regulation, different individuals in the population may show different susceptibility to the disease, which is an exciting concept to follow-up in a subsequent study.

While we characterized the biological consequences only for one molecule in this study, as a proof-of-concept, it represents an important breakthrough, implicating for the first time global alternate splicing particularly in the context of *Mtb* infections. To assume how each of the alternate spliced and alternate poly-adenylated transcripts could together regulate the overall outcome of infection looks both challenging and exciting. It provides new opportunities to explore host responses to infection through the window of post-transcriptional regulations.

Identification of some universal splicing code, which remains elusive till date, has been the focus of the intense investigation by several groups [19, 54, 57]. These results may provide newer tools to explore the spliceosome components, assembly, cis-regulatory elements and their perturbation by external factors like virulent bacterial factors. Interestingly, in some recent reports involvement of mycobacterial factors in regulating host epigenetics has been reported [64, 65] and therefore provides sufficient indications that certain bacterial factors may also directly or indirectly influence the splicing and polyadenylation machinery. An interesting point to note here is that out of five different kinds of AS events, RI events are unique as retained introns can greatly influence the folding of nascent polypeptides. A shift in the RI event post-infection could therefore potentially activate the unfolded protein response (UPR), which is now increasingly getting recognized as yet another arm of host innate defense mechanism [66, 67]. We believe further investigations on this line would add an exciting dimension to the host-pathogen interactions during *Mtb* infection of macrophages. We did not identify any potential regulator of global splicing either from the host or the pathogen in this study. Specific splicing factors are increasingly getting recognized for their anti-cancer properties [63]. Thus there appears enormous opportunity to identify new targets, both from the host and pathogen, for developing potential anti-TB drugs.

In conclusion, we report here global perturbation of alternate splicing and alternate polyadenylation upon *Mtb* infection in the macrophages. As is the case with other known perturbations like regulation of autophagy, apoptosis, gene regulation, phagosome maturation arrest etc. [5, 41, 42, 53, 68], *Mtb* is also able to extract benefit out of the key cellular function of alternate splicing. A better understanding of how *Mtb* can achieve these regulations could result in designing novel approaches for intervention.

## Materials and methods

### Ethics statement

All experiments involving human primary macrophages from healthy volunteers was approved by the Institutional Ethics Committee (approval number: ICGEB/IEC/2016/03)

### Cells, bacterial strains and infection

THP-1 derived macrophages were obtained by treating THP-1 cells with 32 nM PMA in 10% FBS in RPMI 1640 for 24 hours followed by its removal and another 24 hours in 10% FBS in RPMI1640. The THP-1 were then infected with H37Ra and H37Rv strains of Mtb for 4 hours in 10% FBS in antibiotic free RPMI 1640 followed by plain RPMI wash and another 2 hours in 200 μg/ml amikacin to kill any leftover extracellular bacilli. Amikacin was then washed and the cells were kept in 10% FBS in antibiotic free RPMI 1640 for the indicated time points. Media was replaced after every 24 hours.

### Primers

See the S10 Table.

### Total RNA isolation

RNA was extracted from *Mycobacterium tuberculosis* infected THP1 cells using MDI RNA Miniprep kit (MTRK250) according to manufacturer’s guidelines.

### Transcriptome sequencing using HiSEQ2000 platform

Briefly, 100 ng of total RNA was used to prepare amplified cDNA using Illumina TruSeq Kit as per Manufacturer recommended protocol. The produced double-stranded cDNA was subsequently used as the input to the Illumina library preparation protocol starting with the standard end-repair step. The end-repaired DNA with a single ‘A’- base overhang is ligated to the adaptors in a standard ligation reaction using T4 DNA ligase and 2 μM-4 μM final adaptor concentration, depending on the DNA yield following purification after the addition of the ‘A’-base. Following ligation, the samples were purified and subjected to size selection via gel electrophoresis to isolate 350 bp fragments for amplification in preparation for cluster generation. The prepared cDNA library was sequenced for 101-bp paired-end reads using the Hiseq 2000 platform. The image analysis, base calling and quality score calibration were processed using the Illumina Pipeline Software v1.4.1 according to the manufacturer’s instructions. Reads were exported in FASTQ format and has been deposited at the NCBI Sequence Read Archive (SRA) under accession number SRA047025.

### RNA-seq read alignment

Paired end RNA seq reads each of 101bp length from each time point were mapped independently against human genome build hg19 downloaded from Ensemble (http://asia.ensembl.org) using tophat version 2.0.12 (http://tophat.cbcb.umd.edu) with the following options “-p 24 -G Human_ENSEMBL_Coding.gtf ˮ where Human_ENSEMBL_Coding.gtf contains the Ensemble coding transcripts in GTF file format. No novel junctions or novel insertion-deletion were taken in account by passing the parameter “-no-novel-juncˮ and “-no-novel-indelˮ respectively.

### Quantitative expression analysis

Gene and isoform level expression were calculated by using isoform expression method by running cuffdiff (http://cufflinks.cbcb.umd.edu/) on the alignment files from tophat and Ensemble coding genes. For isoforms, multiple mapping reads and sequence bias were corrected by using “-uˮ option of cuffdiff. FPKM (fragments per kilobase of transcript per million mapped read) was used to normalize the variability in number of reads produced for each run and RNA fragmentation during library construction. The count variance was modeled as non linear function of mean counts using negative binomial distribution.

### Analysis of alternate splicing

Differential exon splicing pattern and inclusion level was modeled for each sample compared to uninfected sample using robust Bayesian statistical framework MATS (multivariate analysis of transcript splicing). Exon-exon junction database was constructed from the Ensembl transcript annotation (release 64) file to be used in MATS.A threshold of 0.5 was taken as cutoff to identify significant inclusion level of exons between the samples. Switch like events were considered where inclusion level difference was perfect 1. Further p-value and FDR (False discovery rate) were determined by Markov chain Monte Carlo (MCMC) method by simulating over samples. The quantitative visualization of splice junction of the genes showing significant psi score difference was done in IGV (Integrated genomic viewer) along with uninfected sample as sashimi plot.

### DaPars analysis

Dynamic APA (alternative polyadenylation) usage by each sample compared to uninfected sample was identified by the DaPars (De novo identification of dynamic APA) algorithm. To explain the localized read density change DaPars employs a linear regression model to determine the optimal fitting point. Alignment files were converted to wig file format using RSEM software. PDUI (percentage distal usage index) difference of 0.3 with FDR < 0.05 which passed the filter were considered as significant. The filtered result was visualized using integrated genomic viewer software.

### Q-PCR validations

cDNA was made with iScript cDNA synthesis kit (BioRad #170–8891) using mix of random hexamers and oligo-dT primers. qRT-PCR was performed using SsoFast EvaGreen Supermix (BioRad #1725201) and transcript expression were normalized to actin or 18s rRNA. A list of all primers used for RT-PCR in this study is provided in S10 Table.

### Western blots

1.5X10^6^ cells per well were plated for western blotting experiments. After mentioned time point, cells were washed with ice cold PBS before their incubation with Buffer A solution (20mM HEPES, 10mM NaCl, 1.5mM MgCl_2_, 0.2mM EDTA and 0.5%v/v Trition-X-100) with 1X Protease Arrest (G-Biosciences) for 15 minutes on ice for lysis. Cell lysate was centrifuged at 4°C at 6000g for 10 minutes and supernatant was collected. Protein quantification was done using BSA as standard in Bradford’s assay. Protein sample was mixed with 6X loading dye and subjected to SDS PAGE and transferred to nitrocellulose membrane for immunoblotting. Blocking was done for an hour with Odessey blocking buffer (LI-COR Biosciences) in 1:1 dilution with 1X PBS at room temperature. Blots were immunoblotted with primary and then with secondary antibody made in blocking buffer. Blots were imaged with Odessey Infra Red Imaging system(LI-COR Biosciences).

### Constructs and cloning

Long (ENST00000321437) and short (ENST00000558990) form of Rab8B transcript were amplified from the cDNA and cloned in pMSCV PIG(Puro IRES GFP) (www.addgene.org/21654) between XhoI and EcoRI site of MCS.

### Transfections

HEK293T cells were maintained at 30–50% confluency overnight before transfection in complete 10% FBS DMEM media at 5% CO_2_ and 37^0^ C in a humidified chamber. Lentiviral transfer plasmid was mixed with two packaging plasmid encoding Gag, Pol and other Rev genes in jetPRIME buffer (Polyplus #712–60) in the ratio 3:1:1. Final transfection mix was prepared by adding jetPRIME at a concentration of 1μl/μg DNA, vortexed for 10 seconds and incubated for 10 minutes at room temperature. Transfection mix was added dropwise and incubated for 48 hours before lentiviral harvest. Supernatant was filtered with 0.45μm filter and concentrated using Retro-X concentrator (Clontech #631456).

### Transduction

1X10^6^ THP1 cells were seeded in each well of 6-well plate in 500μl complete RPMI 1640 media and transduced by lentiviral vectors at a multiplicity of infection of 10:1 in the presence of 8μg/ml polybrene. Spinoculation was done for 1 hour at 1200g. Media was replenished with complete media after 6 hours of transduction.

### Microscopy

For immunofluorescence experiments 20X10^4^ cells were plated on autoclaved coverslips one per well in a 24 well plate. Cells were infected with PKH67 labeled or GFP expressing bacteria. The cells were fixed at their requisite time points using 4% v/v paraformaldehyde in 1X PBS for 20 minutes at room temperature. The cells were then incubated with blocking buffer for immunostaining (0.02% Triton-X100, 3% w/v BSA in 1X PBS) for 1 hour. The cells were then washed thrice with 1X PBS for 5 minutes each and incubated with 1:100 anti-Rab8B antibody in blocking buffer for 3 hours. Cells were washed twice with 1X PBS each for 5 minutes and treated with 1:300 anti-rabbit 568nm for 1 hour. The cells were then washed thrice with 1X PBS for 5 minutes each before mounting the coverslips on slides with Pro-Long anti-fade reagent (Life Technologies). All the incubation and staining was done at room temperature. NIS-Elements (Nikon) was used for acquiring the images as Z-stacks using Nikon EclipseTi-E laser scanning confocal microscope equipped with a 60X/1.4 NA Plan Apochromat DIC objective.

### Flow cytometry

For FACS experiments 1 X 10^6^ transduced THP1 macrophages were plated per well in a 6 well plate and infected with Far-red labeled bacteria. The time and staining concentrations of Far-red dye was performed as per manufacturer’s directions. The cells were fixed at their requisite time points using 4% v/v paraformaldehyde in 1X PBS for 20 minutes at room temperature. Cells were scrapped and data was acquired using BD FACSDiva acquisition software in BD FACS Canto II flow cytometer (BD). Data was analyzed and plotted using R packages flowcore and flowViz.

### Primary human PBMC-derived macrophages

Heparinized blood was immediately diluted in 1:1 ratio by volume with DPBS. Diluted blood was layered on Ficoll-paque (Himedia) and centrifuged at 2000 rpm for 45 min. Interface containing PBMC was isolated carefully and washed twice with DPBS. Cells were diluted in RPMI1640 media containing 10% FBS to a concentration of 1x10^6^ cells/ml. Cells were put in a 6 well tissue culture plate and incubated for 3 hours in a humidified 5% co_2_ chamber at 37^°^C. Non adherent cells were removed and two washes with RPMI was done. Complete media containing 5ng/ml recombinant human M-CSF (R&Dsystems, 216-MC/CF) was added and cells were allowed to differentiate for 4 days into macrophages in a humidified 5% co_2_ chamber at 37°C.

### siRNA transfection

siGenome human Rab8B siRNA (SmartPool of 4 different siRNA, M-008744-01-0005) was obtained from Dharmacon (Dharmacon, GE Healthcare). Cells were transfected at final concentration of 50nM siRNA using Dharmafect-2 transfection reagent according to manufacturer's protocol.

### CFU determination

After indicated time period cells were lysed in 50ul of 0.06% SDS containing lysis buffer at room temperature for 10 min. 1:10 and 1:50 dilution of lysate was prepared and plated in duplicates on 7H11 agar plates supplemented with 10% OADC. Square plates (12cm x 12 cm) were used for plating by track dilution method. 10μl of the diluted sample was spotted and allowed to flow by tilting the plate at 45^0^ angle. Plates were dried and incubated for 18–20 days in a humidified incubator at 37°C before colonies were counted.

## Supporting information

S1 Fig Reads dispersion and median FPKM distribution at gene level across the samples. (A) The dispersion of alignment of reads obtained at gene level in each sample is plotted here.
(B) Distribution of Log_10_FPKM around the median within each sample was plotted.
(PDF)

S2 Fig Density distribution of gene level FPKM. Density distribution plots against FPKM for each sample overlaid on the uninfected control sample.
(PDF)

S3 Fig Density distribution at isoform level FPKM and junction reads for AS. (A) Density distribution plots against FPKM calculated at the isoform level for each sample overlaid on the uninfected control sample.
(B) Density plots for junction reads in H37Ra and H37Rv infected macrophages across the course of infection.
(PDF)

S4 Fig AS cases for genes that were differentially regulated upon infection. The six plots here are same as the plots in Fig 3B except that in this case only those genes, which showed significant AS as well as significant regulation at gene level were plotted for their psi-scores. Thus they actually correspond to the numbers shown in Fig 3D. Six plots refer to six different time points post-infection.
(PDF)

S5 Fig No. of transcripts with different 3’UTR length and corresponding FPKM. The plot compares UTR length versus FPKM and number of transcripts across each of the sample.
(PDF)

S6 Fig Truncated transcripts of ACSL1 and Dynamin-1. (A) SASHIMI plot for reads alignment at ACSL1 locus. Number of reads corresponding to specific exon-exon junctions (shown as loops) is labeled for each junction.
(B) The exon organization of ACSL1 isoform studied here. The shorter isoform was validated using RTPCR. Each colored block corresponds to one exon. Arrows identify sites for primer binding.
(C) Gene level quantification of ACSL1 expression in UI, H37Ra infected or H37Rv infected THP-1 macrophages
(D) Percent contribution of ACSL1 transcripts across the three groups. The red bar corresponds to the shorter transcript analyzed in this study.
(E) Fold expression of ACSL1 shorter transcript validated by Q-PCR in an independent set of experiments.
(F) SASHIMI plot for reads alignment at Dynamin-1 locus. Number of reads corresponding to specific exon-exon junctions (shown as loops) is labeled for each junction.
(G) The exon organization of Dynamin-1 isoform studied here. The shorter isoform was validated using RTPCR. Each colored block corresponds to one exon. Arrows identify sites for primer binding.
(G) Gene level quantification of Dynamin-1 expression in UI, H37Ra infected or H37Rv infected THP-1 macrophages
(I) Percent contribution of Dynamin-1 transcripts across the three groups. The red bar corresponds to the shorter transcript analyzed in this study.
(J) Fold expression of Dynamin-1 shorter transcript validated by Q-PCR in an independent set of experiments.
(PDF)

S7 Fig Truncated transcripts influence macrophage response to infection. (A) THP-1 macrophages infected with H37Ra, H37Rv or infected were lysed at 48 hours post-infection and Western blot against ACSL1 protein was performed.
(B) THP-1 macrophages were transduced with vector control, RAB8B long or RAB8B short constructs. Cells were infected with H37Ra or H37Rv as described in Fig 7F. In addition to CFU plating for Fig 7F, we in parallel acquired the cells through flow cytometry to estimate percent cells that were transduced. Data from RAB8B long isoform transduced cells are shown here in the Fig.
(C) THP-1 macrophages were transduced with vector control, RAB8B long or RAB8B short constructs. Cells were infected with H37Rv and at 48 hours post-infection, cells were harvested and Western blot for RAB8B was performed (UI: uninfected control).
(D) siRNA-mediated knockdown of RAB8B in primary MDMs. We followed the standard protocol as standardized in THP- macrophages for siRNA-mediated knockdown. At 48 hours post siRNA treatment, cells were lysed and RAB8B Western blot was performed.
(PDF)

S1 Table Scores for QC and alignment statistics. (XLSX)

S2 Table Gene expression profile and corresponding p-values. (XLSX)

S3 Table GO analysis of differentially regulated genes. (XLSX)

S4 Table Transcript expression profile and corresponding p-values. (XLSX)

S5 Table Psi-score for transcripts along with p-values. (XLSX)

S6 Table GO analysis of genes undergoing alternate splicing. (XLSX)

S7 Table APA list identified through DAPARS. (XLSX)

S8 Table GO analysis of genes undergoing APA. (XLSX)

S9 Table Transcript expression profiles for genes associated with spliceosome. (XLSX)

S10 Table List of primers. (XLSX)
