# Correction: Alternate splicing of transcripts shape macrophage response to *Mycobacterium tuberculosis* infection

**DOI:** 10.1371/journal.ppat.1006833

**Published:** 2018-01-04

**Authors:** Haroon Kalam, Mary F. Fontana, Dhiraj Kumar

The Data Availability statement for this paper is incorrect. The correct statement is: Data is available at NCBI sequence read archive (SRA) under accession number SRP125165.
