# Supplementary figures and images for: Alternate splicing of transcripts shape macrophage response to Mycobacterium tuberculosis infection
Source: PLoS Pathog. 2017 Mar 3;13(3):e1006236. doi: 10.1371/journal.ppat.1006236 (PMC5352146; doi:10.1371/journal.ppat.1006236)

Figure S1

A

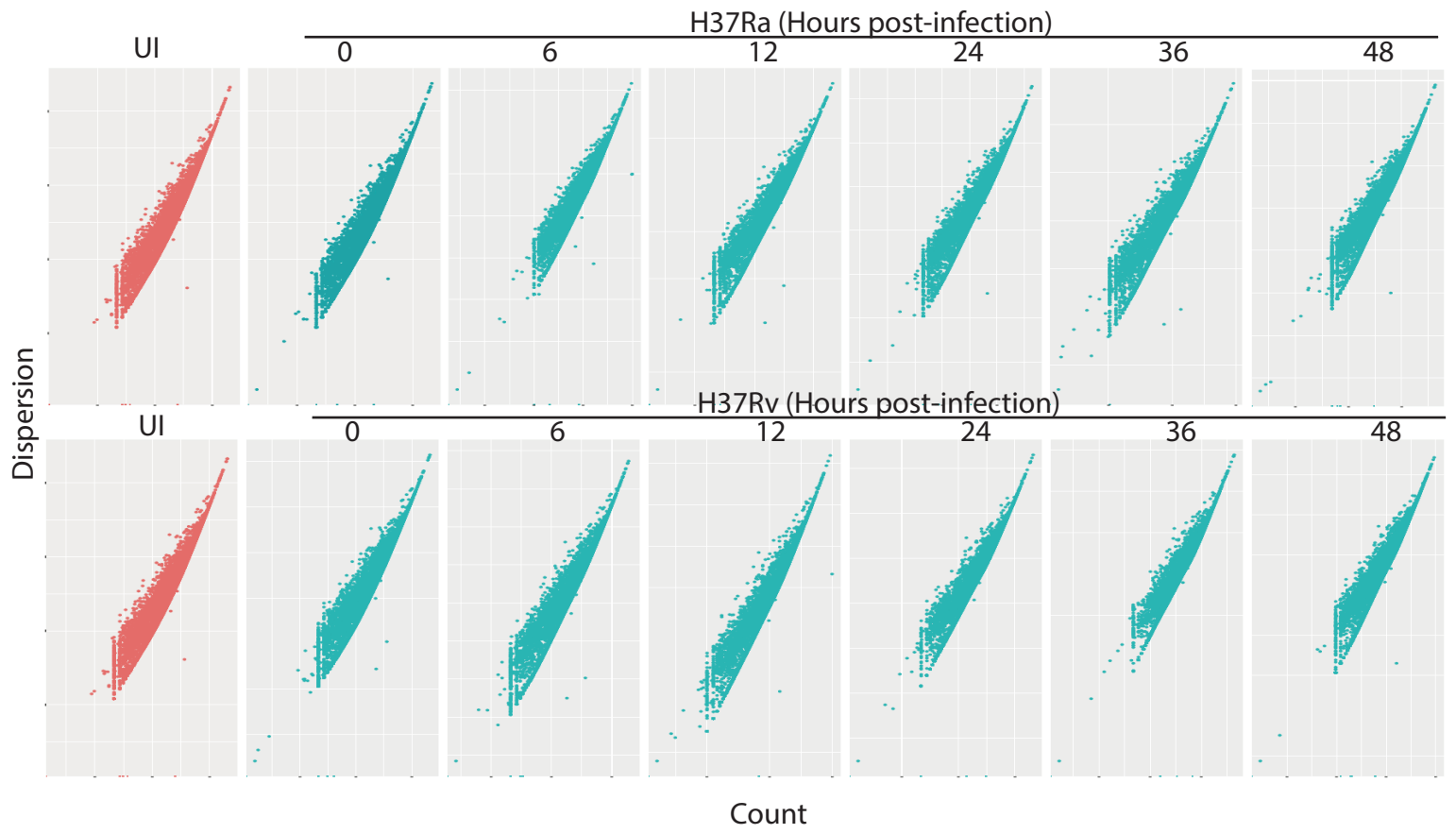

B

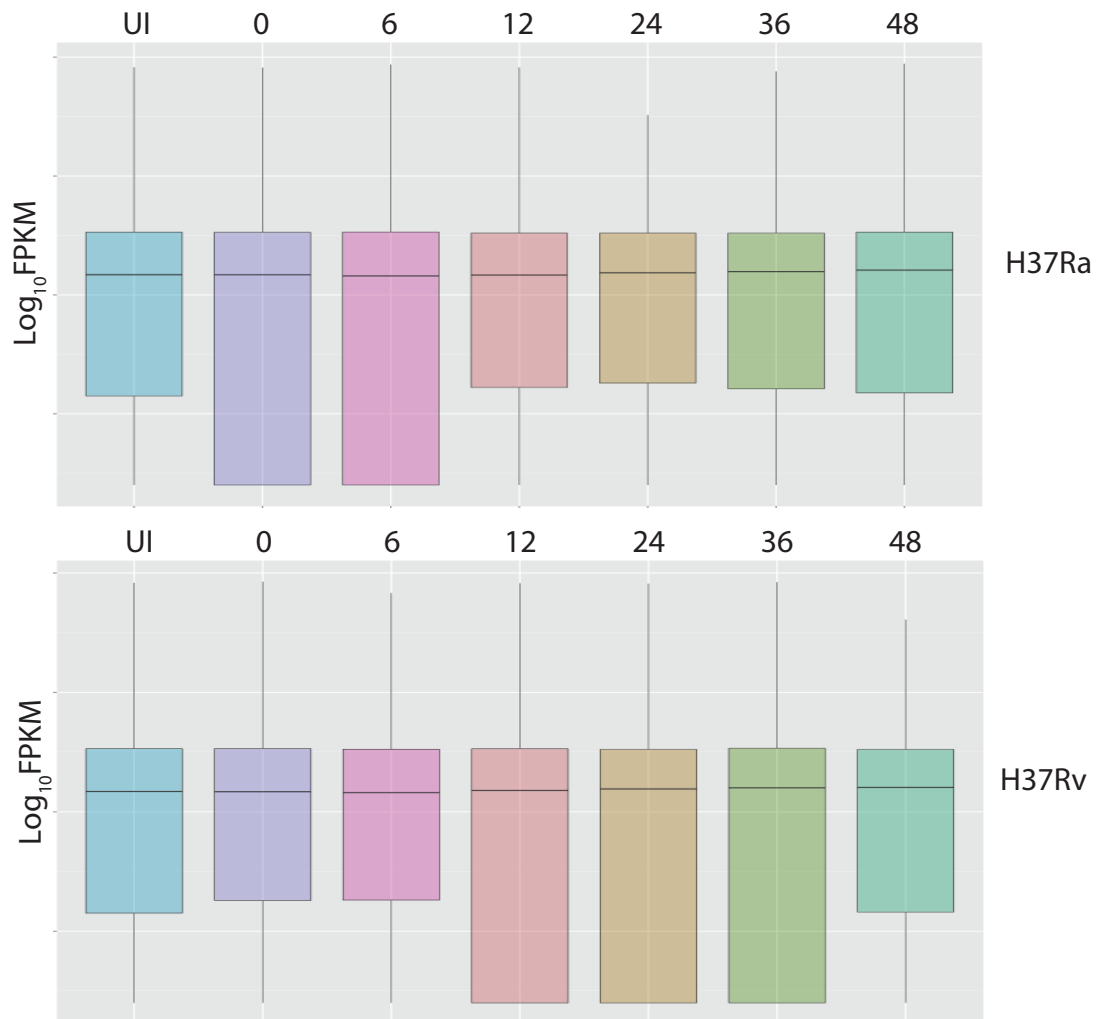

Supplement: S1 Fig — (A) The dispersion of alignment of reads obtained at gene level in each sample is plotted here. (B) Distribution of Log10FPKM around the median within each sample was plotted. (PDF) [file ppat.1006236.s001.pdf]

Figure S2

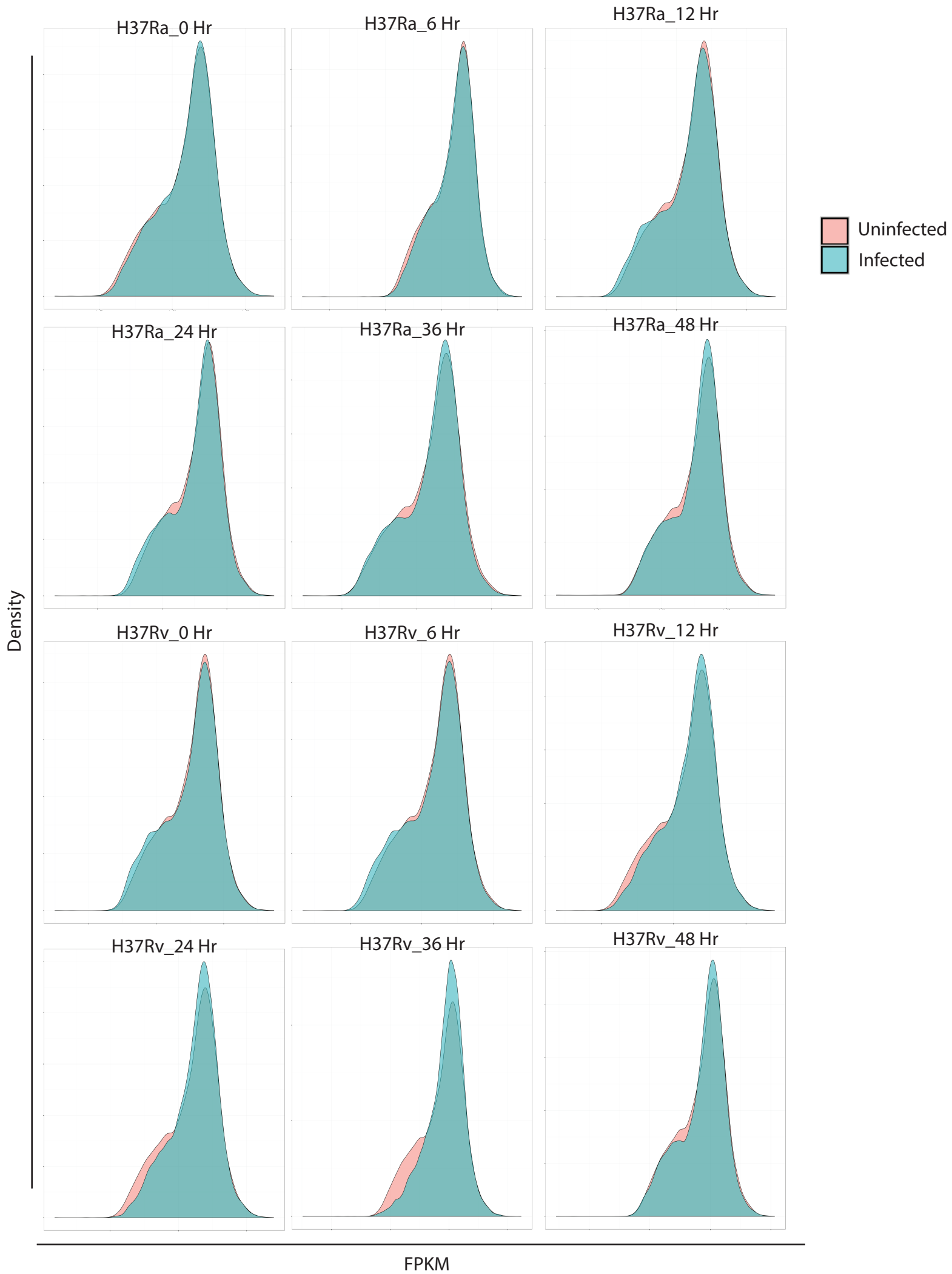

Supplement: S2 Fig — Density distribution plots against FPKM for each sample overlaid on the uninfected control sample. (PDF) [file ppat.1006236.s002.pdf]

Figure S3

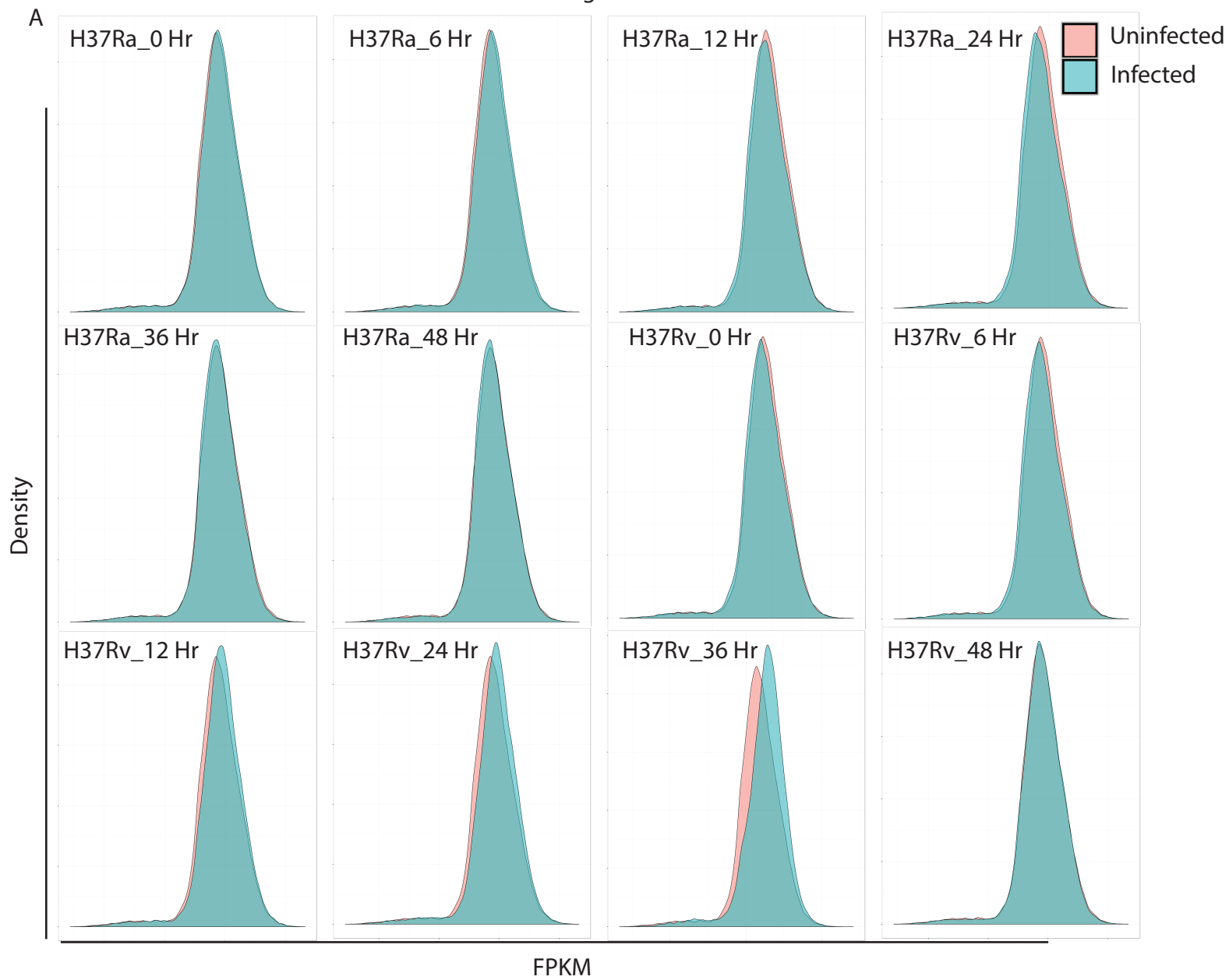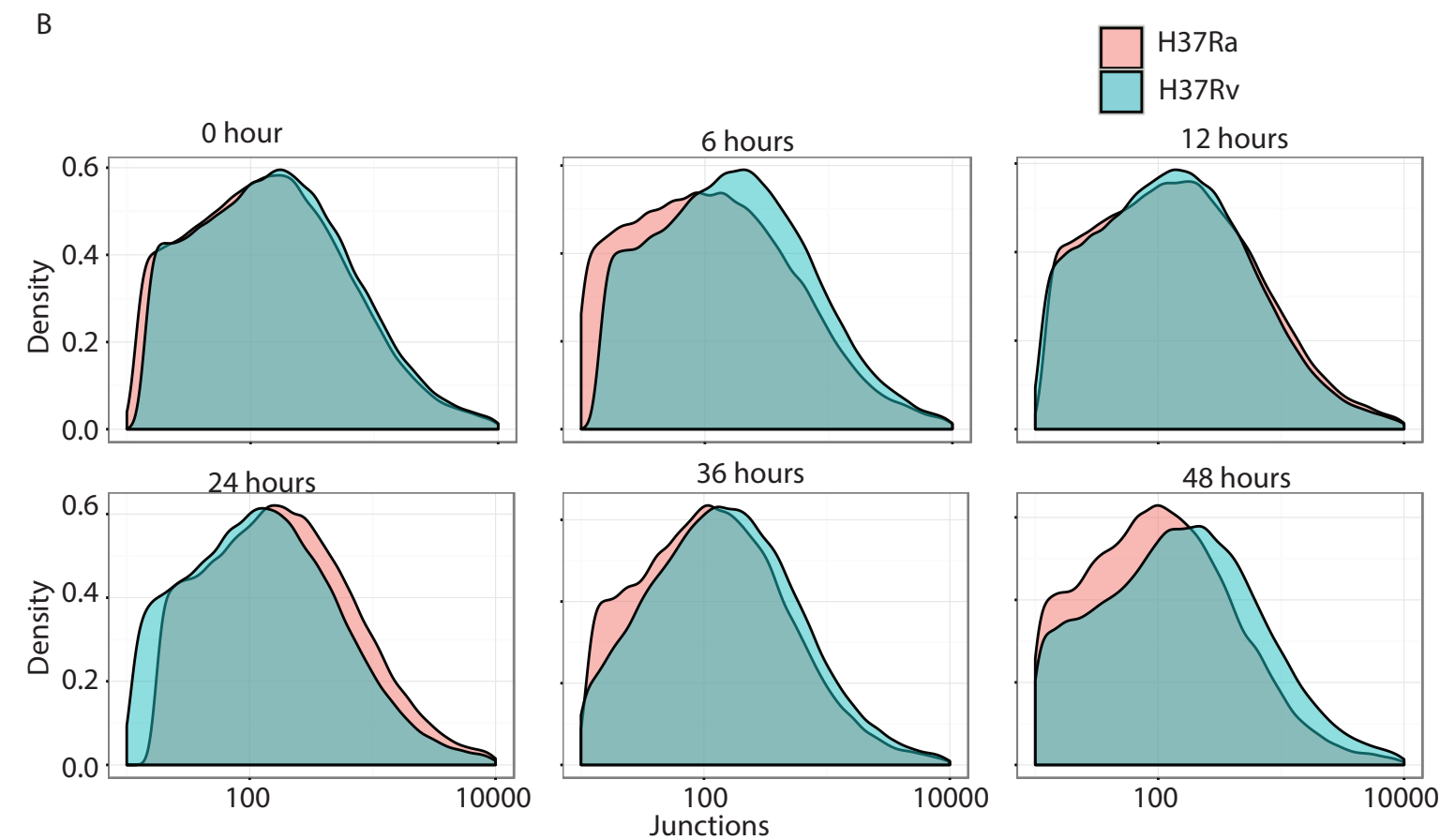

Supplement: S3 Fig — (A) Density distribution plots against FPKM calculated at the isoform level for each sample overlaid on the uninfected control sample. (B) Density plots for junction reads in H37Ra and H37Rv infected macrophages across the course of infection. (PDF) [file ppat.1006236.s003.pdf]

Figure S4

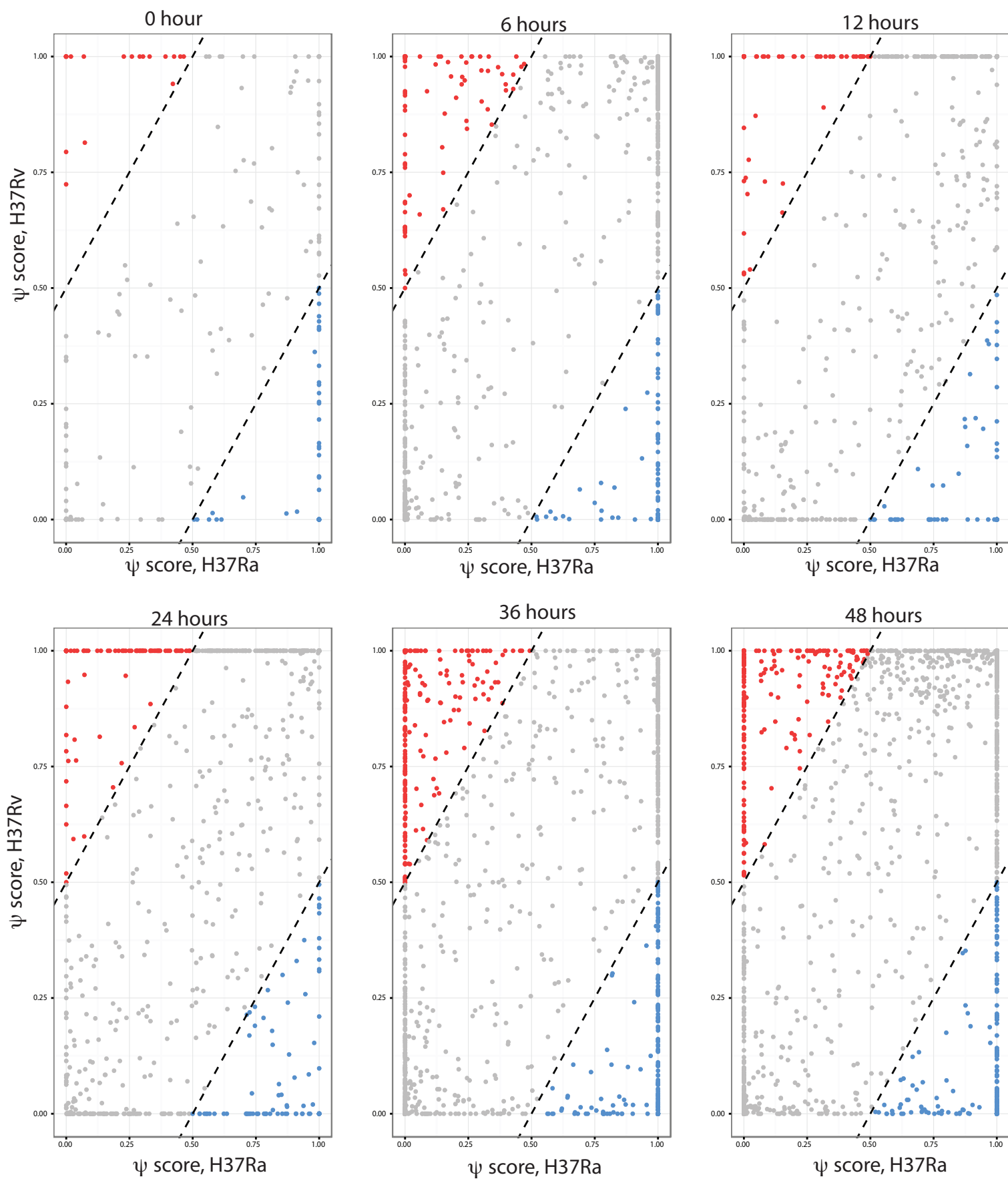

Supplement: S4 Fig — The six plots here are same as the plots in Fig 3B except that in this case only those genes, which showed significant AS as well as significant regulation at gene level were plotted for their psi-scores. Thus they actually correspond to the numbers shown in Fig 3D. Six plots refer to six different time points post-infection. (PDF) [file ppat.1006236.s004.pdf]

Figure S5

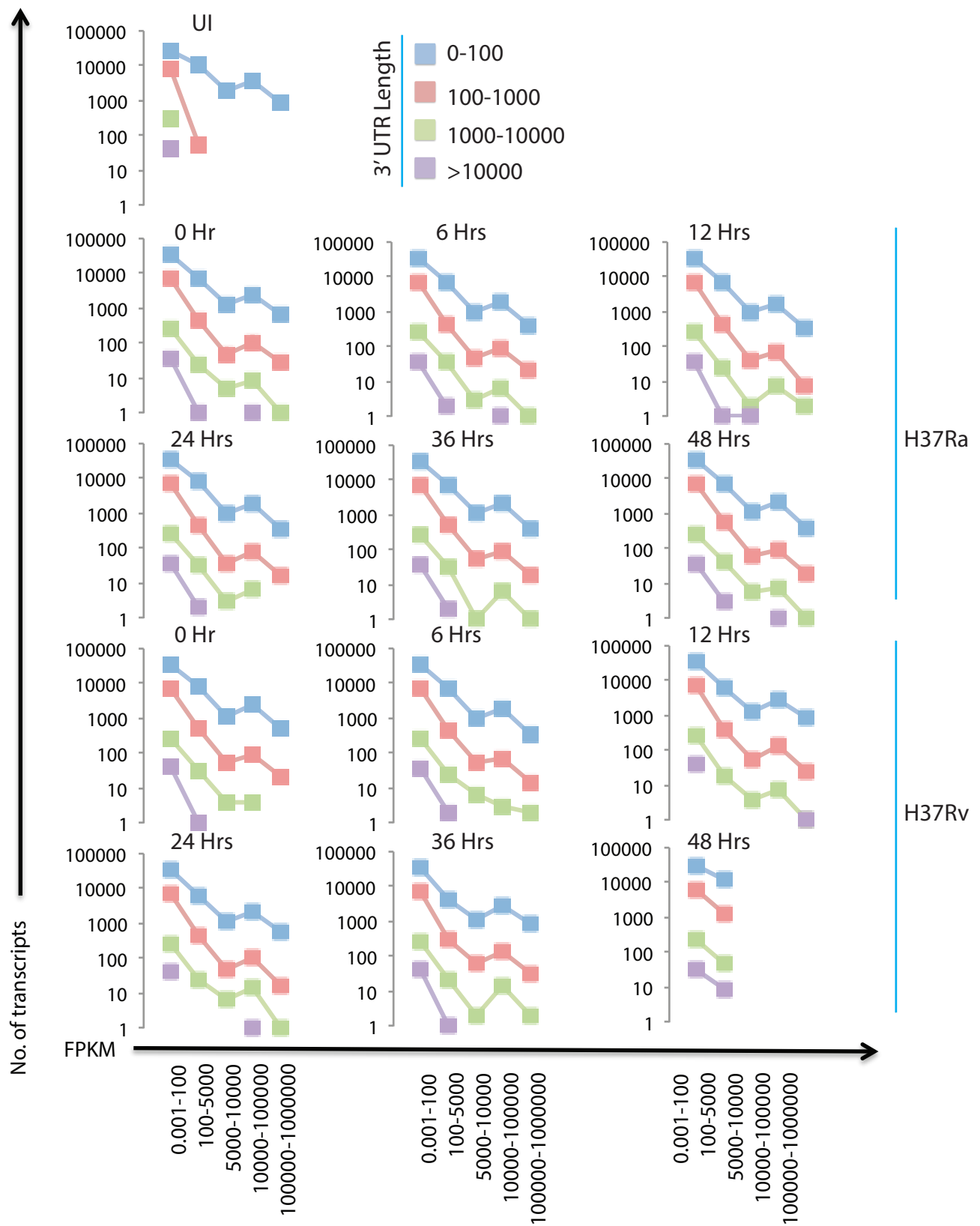

Supplement: S5 Fig — The plot compares UTR length versus FPKM and number of transcripts across each of the sample. (PDF) [file ppat.1006236.s005.pdf]

Figure S6

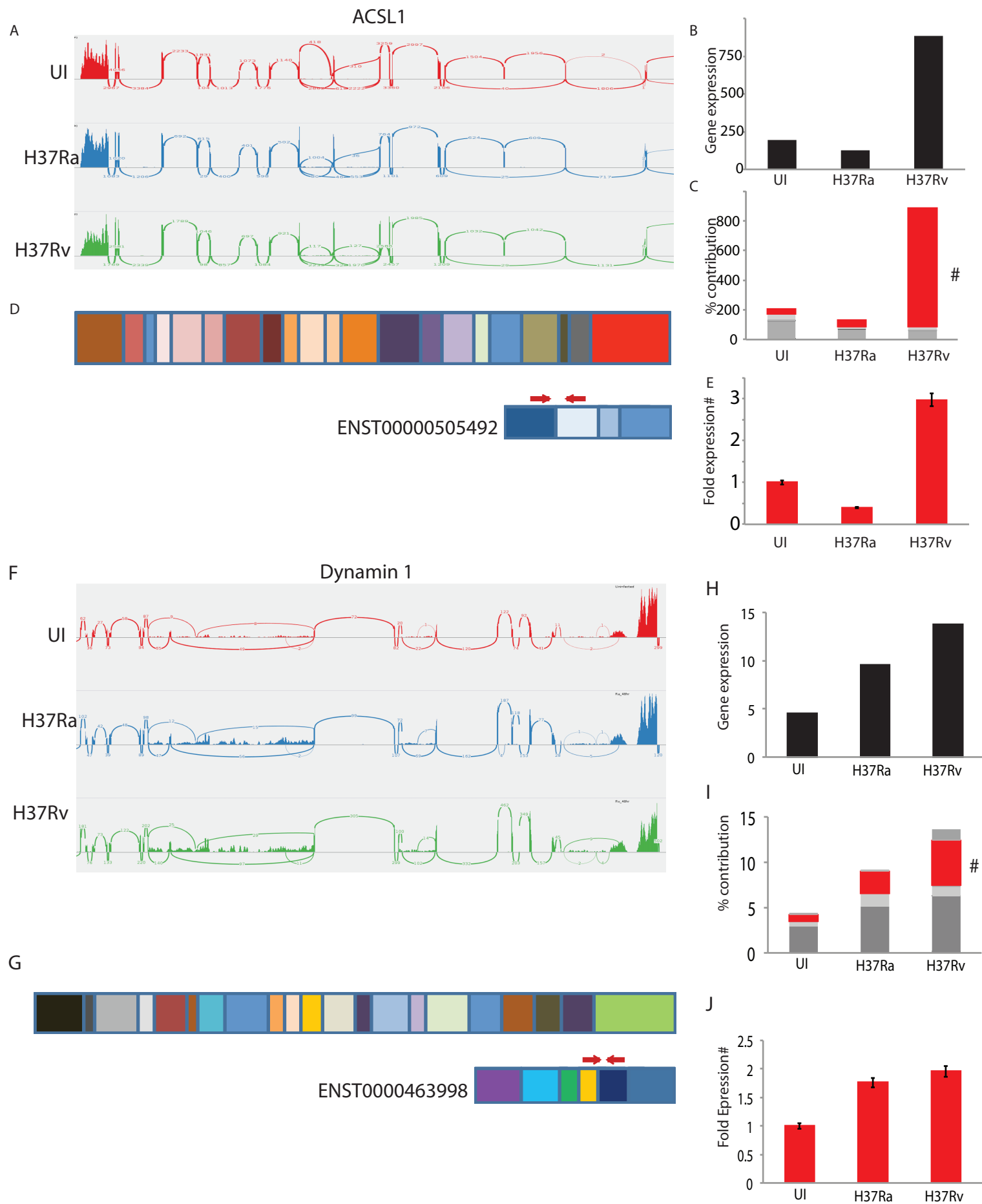

Supplement: S6 Fig — (A) SASHIMI plot for reads alignment at ACSL1 locus. Number of reads corresponding to specific exon-exon junctions (shown as loops) is labeled for each junction. (B) The exon organization of ACSL1 isoform studied here. The shorter isoform was validated using RTPCR. Each colored block corresponds to one exon. Arrows identify sites for primer binding. (C) Gene level quantification of ACSL1 expression in UI, H37Ra infected or H37Rv infected THP-1 macrophages (D) Percent contribution of ACSL1 transcripts across the three groups. The red bar corresponds to the shorter transcript analyzed in this study. (E) Fold expression of ACSL1 shorter transcript validated by Q-PCR in an independent set of experiments. (F) SASHIMI plot for reads alignment at Dynamin-1 locus. Number of reads corresponding to specific exon-exon junctions (shown as loops) is labeled for each junction. (G) The exon organization of Dynamin-1 isoform studied here. The shorter isoform was validated using RTPCR. Each colored block corresponds to one exon. Arrows identify sites for primer binding. (G) Gene level quantification of Dynamin-1 expression in UI, H37Ra infected or H37Rv infected THP-1 macrophages (I) Percent contribution of Dynamin-1 transcripts across the three groups. The red bar corresponds to the shorter transcript analyzed in this study. (J) Fold expression of Dynamin-1 shorter transcript validated by Q-PCR in an independent set of experiments. (PDF) [file ppat.1006236.s006.pdf]

Figure S7

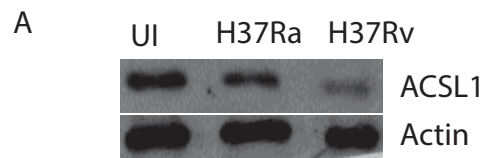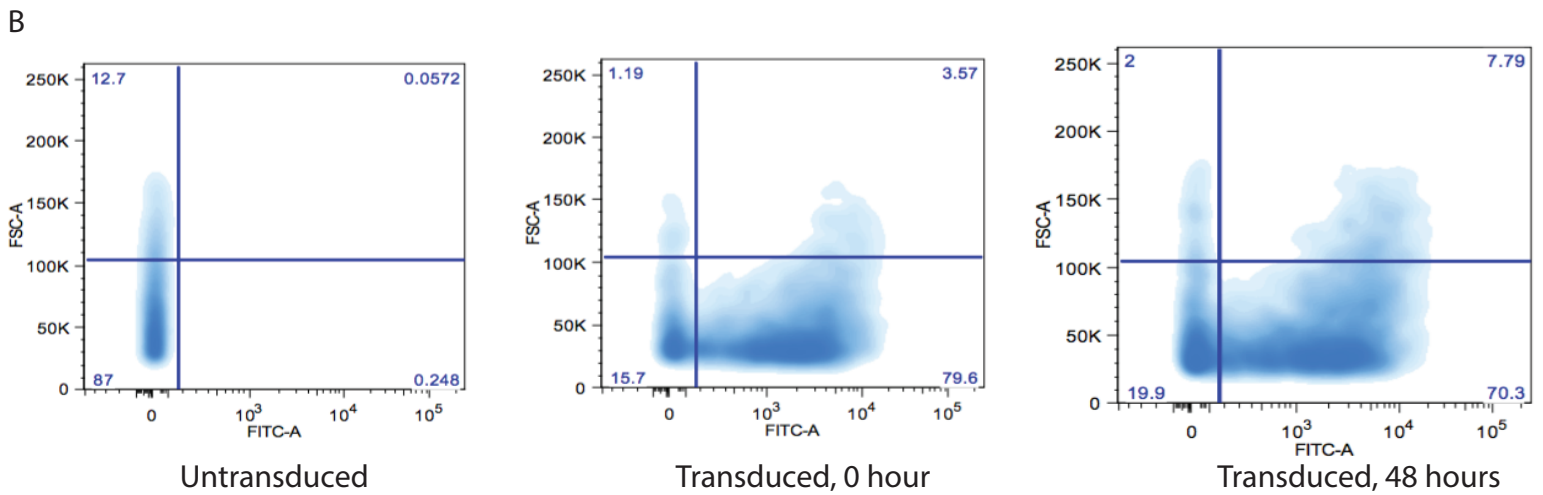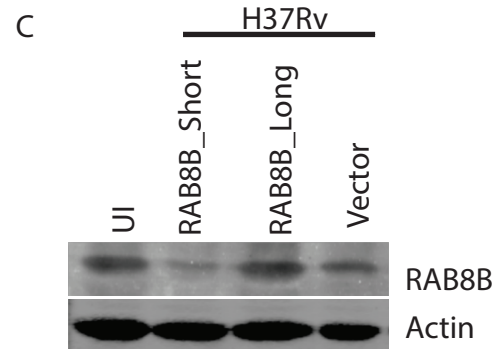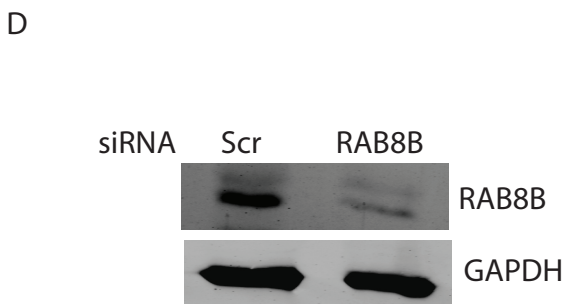

Supplement: S7 Fig — (A) THP-1 macrophages infected with H37Ra, H37Rv or infected were lysed at 48 hours post-infection and Western blot against ACSL1 protein was performed. (B) THP-1 macrophages were transduced with vector control, RAB8B long or RAB8B short constructs. Cells were infected with H37Ra or H37Rv as described in Fig 7F. In addition to CFU plating for Fig 7F, we in parallel acquired the cells through flow cytometry to estimate percent cells that were transduced. Data from RAB8B long isoform transduced cells are shown here in the Fig. (C) THP-1 macrophages were transduced with vector control, RAB8B long or RAB8B short constructs. Cells were infected with H37Rv and at 48 hours post-infection, cells were harvested and Western blot for RAB8B was performed (UI: uninfected control). (D) siRNA-mediated knockdown of RAB8B in primary MDMs. We followed the standard protocol as standardized in THP- macrophages for siRNA-mediated knockdown. At 48 hours post siRNA treatment, cells were lysed and RAB8B Western blot was performed. (PDF) [file ppat.1006236.s007.pdf]
